# The march of pluripotent stem cells in cardiovascular regenerative medicine

**DOI:** 10.1186/s13287-018-0947-5

**Published:** 2018-07-27

**Authors:** Haissam Abou-Saleh, Fouad A. Zouein, Ahmed El-Yazbi, Despina Sanoudou, Christophe Raynaud, Christopher Rao, Gianfranco Pintus, Hassan Dehaini, Ali H. Eid

**Affiliations:** 1 Department of Biological and Environmental Sciences, Qatar University, Doha, Qatar; 2 Department of Pharmacology and Toxicology, Faculty of Medicine, American University of Beirut, Beirut, Lebanon; 3 Department of Biomedical Sciences, College of Health Sciences, Qatar University, Doha, Qatar; 4 Department of Pharmacology and Toxicology, Alexandria University, Alexandria, Egypt; 5 Clinical Genomics and Pharmacogenomics Unit, 4th Department of Internal Medicine, “Attikon” Hospital, Medical School, National and Kapodistrian University of Athens, Athens, Greece; 6 Sidra Medical and Research Center, Doha, Qatar; 7 Department of Surgery, Queen Elizabeth Hospital, Woolwich, London, UK

**Keywords:** Cardiovascular disease, Stem cell therapy, iPSCs, Heart failure, Cardiomyocytes, Regenerative medicine

## Abstract

Cardiovascular disease (CVD) continues to be the leading cause of global morbidity and mortality. Heart failure remains a major contributor to this mortality. Despite major therapeutic advances over the past decades, a better understanding of molecular and cellular mechanisms of CVD as well as improved therapeutic strategies for the management or treatment of heart failure are increasingly needed. Loss of myocardium is a major driver of heart failure. An attractive approach that appears to provide promising results in reducing cardiac degeneration is stem cell therapy (SCT). In this review, we describe different types of stem cells, including embryonic and adult stem cells, and we provide a detailed discussion of the properties of induced pluripotent stem cells (iPSCs). We also present and critically discuss the key methods used for converting somatic cells to pluripotent cells and iPSCs to cardiomyocytes (CMs), along with their advantages and limitations. Integrating and non-integrating reprogramming methods as well as characterization of iPSCs and iPSC-derived CMs are discussed. Furthermore, we critically present various methods of differentiating iPSCs to CMs. The value of iPSC-CMs in regenerative medicine as well as myocardial disease modeling and cardiac regeneration are emphasized.

## Background

Cardiovascular disease (CVD) remains the leading cause of death worldwide, killing 17 million people each year. The World Health Organization (WHO) estimates that by 2020 this number will reach 24 million. With complex multifactorial pathologies, including both genetic and environmental factors, CVD continues to be difficult to prevent. Current strategies against CVD include prevention (i.e., lifestyle changes) and pharmacological and/or surgical intervention. However, the effectiveness of drug treatment varies among individuals, while surgical interventions may not be applicable to all patients. New approaches need to be established to better understand the mechanisms of CVD and improve diagnostic and therapeutic strategies, particularly in the context of heart failure.

Loss of myocardium results in the clinical syndrome of heart failure [1]. The long-term prognosis of heart failure is poor and current therapies are largely palliative [2, 3]. The only treatment for end-stage heart failure with established long-term efficacy is transplantation. However, the increasing prevalence of heart failure and existing shortage of donor organs are frequent challenges [4, 5].

Stem cell therapy (SCT) aims to reduce cardiac degeneration by regenerating cardiomyocytes (CMs) and is currently considered one of the most promising therapeutic strategies [6, 7]. Stem cells are undifferentiated cells theoretically capable of renewing themselves indefinitely under appropriate conditions through mitotic cell division, and can maintain, generate, or replace damaged tissue by differentiating into specialized cell types [8]. This review describes different types of stem cells, including embryonic stem cells (ESCs) and adult stem cells (ASCs), and focuses primarily on induced pluripotent stem cells (iPSCs). The key methods used for converting somatic cells to iPSCs and then to CMs are presented, along with their advantages and limitations. Emphasis is given to the value of iPSC-derived CMs (iPSC-CMs) in regenerative medicine and myocardial disease modeling.

## Stem cell potency

Stem cells can be classified according to their “potency” or “differentiation potential” (Table 1). Importantly, newer cell types, such as iPSC-CMs, directly transdifferentiated CMs, and endogenous cardiac stem cell derived CMs (CSC-CMs), could be easily obtained from any individual and used to create patient- and disease-specific models, enabling the elucidation of molecular and genetic mechanisms that underlie inherited diseases phenotypes and unveiling novel therapeutic and personalized therapeutic targets [9–14].

**Table 1:** Differential potential of stem cells

Differential potential	Number of stem types	Original stem cell	Differentiated cells
Totipotential	All	Fertilized egg (zygote)	All cell types
Pluripotential	All except cells of the embryonic membranes	Cultured embryonic stem cells (ESCs)	Cells from all three germ layers
Multipotential	Many	Adult stem cells (bone marrow, cord blood, peripheral blood, heart, lung)	Blood cells, cardiomyocytes, neural cells, hepatocytes, endothelial cells, myocytes
Oligopotential	Few (2–4 cells)	Myeloid precursor, mesenchymal stem cell, glial-restricted precursor	Myeloid cells, stromal cells, osteocytes, chondrocytes, adipocytes
Unipotential	1	Mast cell precursor	Mast cell
Nullipotentail	None	Terminally differentiated cell (e.g., red blood cell)	No cell division

## Multipotent stem cells for SCT

Adult or somatic stem cells (ASCs) are non-embryonic multipotent stem cells found in the adult organism after embryonic development and residing in an area in tissues called the “stem cell niche” [15, 16]. ASCs exist in various tissues, such as the bone marrow [17, 18], cord blood [19, 20], skeletal muscles [21, 22], peripheral blood [23, 24], adipose tissue [25, 26], lung [27, 28], and the heart [29, 30]. Unlike ESCs, ASC origins are not well defined and their multipotency is very limited. Their primary functions are to maintain the homeostasis of mature cell tissues and, with limitations, to regenerate damaged organs. However, ASCs are rare in mature tissues, have limited capacity to differentiate into multiple cell lineages, and behave differently depending on environmental stimuli. In addition, their isolation from adult tissues is challenging, and methods of culture have not yet been optimized. For example, bone marrow-derived hematopoietic stem cells (HSCs) have been studied in multiple diseases, including bone-marrow failure [31], vasculogenesis [32, 33], and cardiac regeneration [17, 34]. However, HSCs represent a very small fraction (only 0.01–0.015%) of the total bone marrow cells and their therapeutic and differentiation potential is highly controversial [35, 36]. Consequently, although ASCs would represent a valuable and promising source of stem cells and SCT, their use is still hindered by a series of biological and technical limitations that require further investigation.

## Pluripotent stem cells for SCT: shift from ESCs to iPSCs

**Fig. 1:**
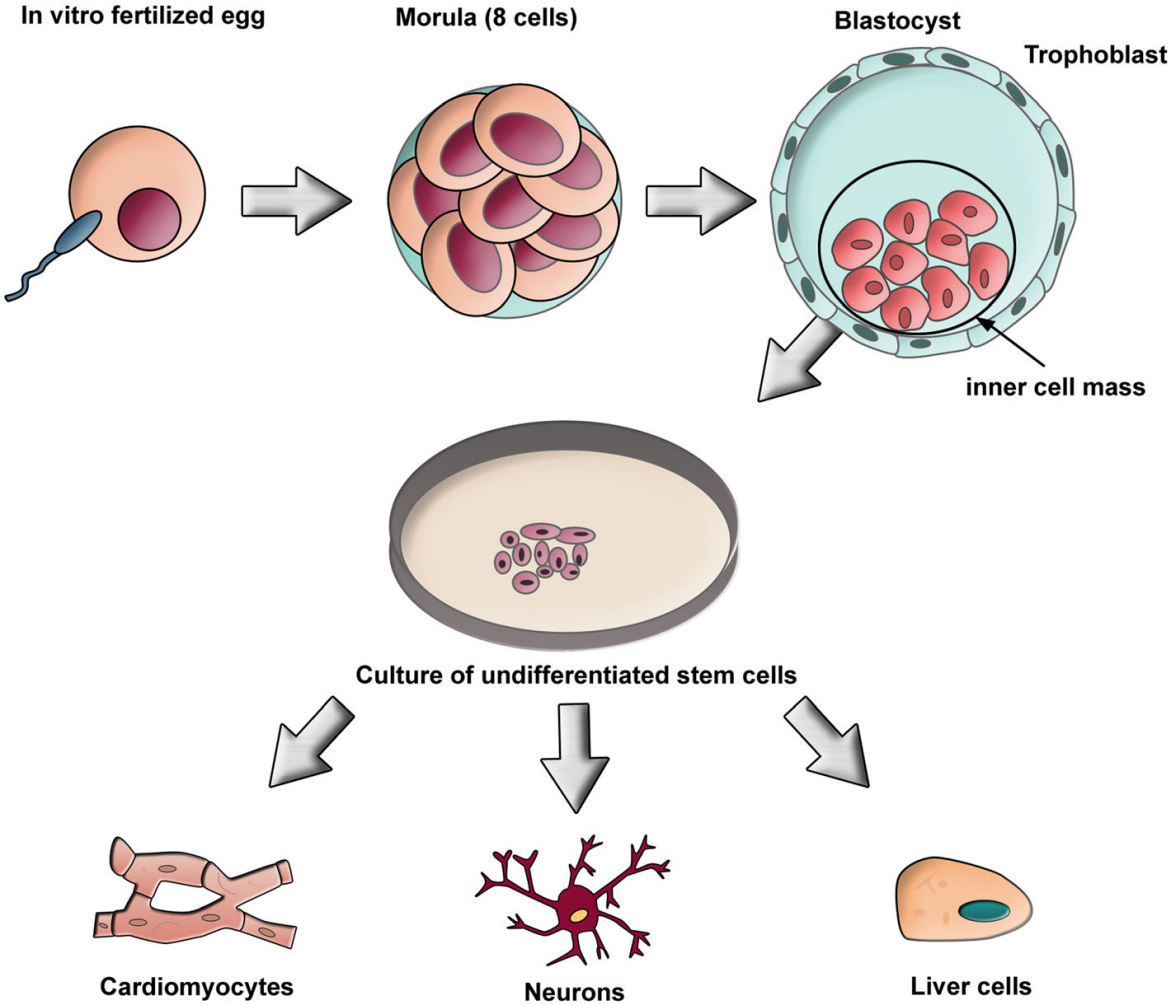
Generation of embryonic stem cells. A fertilized egg is allowed to develop to the blastocyst stage. The inner cell mass dissociates from the trophoblast by laser dissection or enzymatic digestion. Isolated cells are cultured in this pluripotent state for a long period of time in the presence of growth factors. The pluripotent stem cells can be differentiated into various cell lineages, such as cardiomyocytes, neurons, or liver cells

ESCs are isolated from embryos and can be classified as totipotent or pluripotent depending on their temporal existence during fetal development. Totipotent ESCs are present in the earliest eight-cell stage embryo, whereas pluripotent ESCs are found throughout the remainder of embryonic development. In this review, ESCs refer to the pluripotent type of ESCs, obtained from a 4- or 5-day-old embryo, also known as the blastocyst phase of development. ESCs are extracted from the inner cell mass of blastocysts and placed in a controlled culture that allows them to divide indefinitely without further cell differentiation. These ex vivo expanded cells serve as a paramount source of stem cells for transplantation therapies for many diseases, including cardiomyopathies, neurological disorders, and diabetes (Fig. 1). However, a series of ethical and technical issues restricts ESC use [37]. Technically, the use of ESCs for cell transplantation requires a differentiation step to the target cell lineage with formation of undifferentiated cells amongst the cellular product [38]. This can induce spontaneous teratoma formation in host tissue, raising safety concerns that must be carefully addressed [39, 40]. Moreover, the allogeneic nature of ESCs may induce immune responses with a prominent risk of rejection.

Ethically, the use of human ESCs (hESCs) is controversial, with many pro-life advocates being concerned about the isolation of hESCs from “living” embryos. In 2001, the USA government banned stem cell research by restricting federal funding for research on hESCs. To allow responsible scientific research involving human stem cells, the National Institutes of Health (NIH) established the “Human Embryonic Stem Cell Registry”, which lists 177 stem cell lines that are suitable for employment in federally funded research. Unfortunately, not all of these stem cell lines are readily available, and scientists have concerns about the quality and the longevity of these stem cell lines. To bypass these challenges, an increasing number of laboratories around the world are currently using iPSCs to limit the use of hESCs and the destruction of living human embryos.

## iPSCs: the promising era of SCT

Practical considerations such as the availability of embryonic tissues and the isolation of relatively rare cell types have limited the large-scale production of pure stem cells for industrial and clinical applications. As such, the stem cell research field has explored other options, such as transforming fully differentiated adult somatic cells into pluripotent stem cell (PSCs). The reacquisition of a pluripotent state, known as “cell reprogramming”, represents a paradigm shift in our understanding of cellular differentiation and of the plasticity of the differentiated state.

### Historical overview

**Fig. 2:**
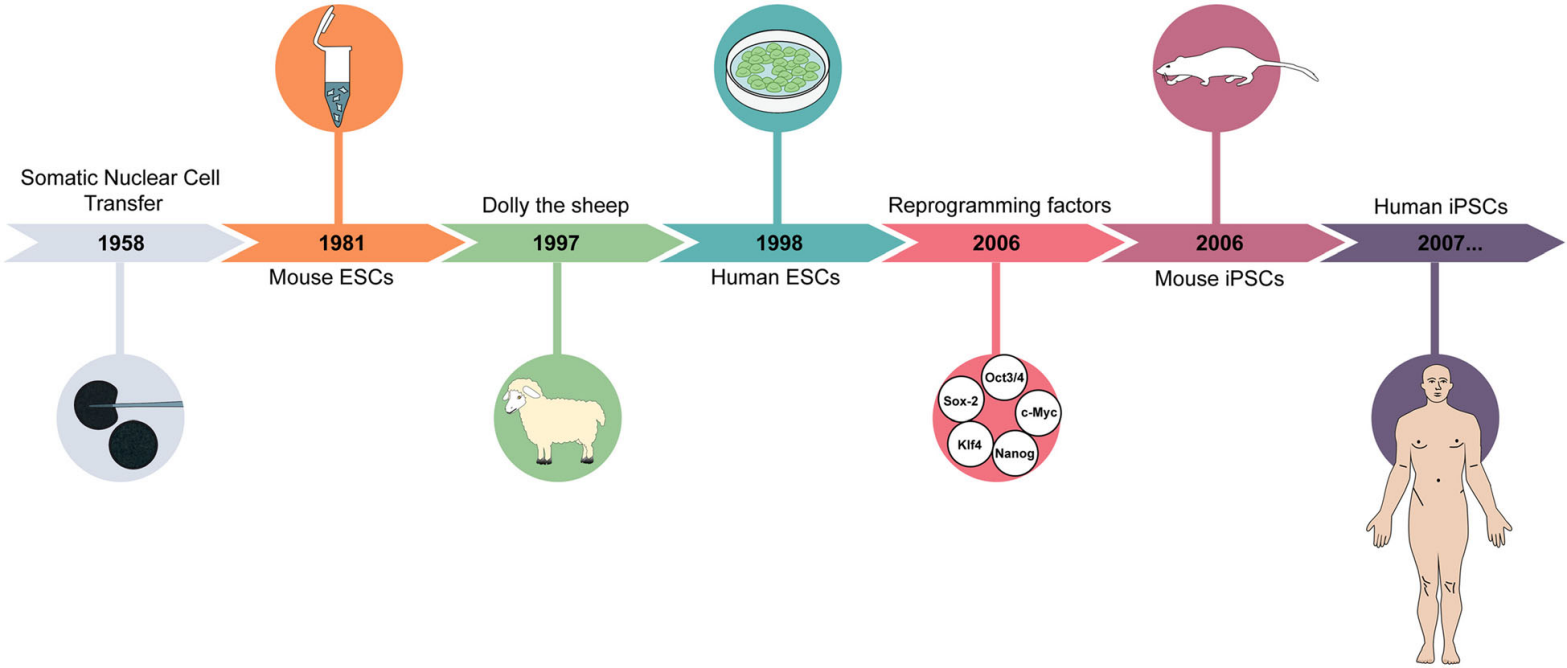
Stem cell research: key dates. Genetic reprogramming started as early as 1958 with the first somatic nuclear cell transfer, demonstrating that the nucleus was responsible for the function of a cell. The derivation of the first embryonic stem cell from mice was only achieved in the early 1980s. The major breakthrough that turned world attention toward cloning and genetic manipulation happened in 1997 with the first animal cloning of the famous sheep Dolly. Soon after, in 1998, the first human embryonic stem cell was derived. Those cells remained the only pluripotent stem cells at the disposal of researchers until 2006, when Shinya Yamanaka identified the reprogramming factors capable of inducing pluripotency in adult cells. Somatic nuclear cell transfer image is courtesy of Howard Hughes Medical Institute (HHMI). Mouse ESC image is courtesy of emouseatlas.org. Dolly the sheep, human ESC, and mouse iPSC images are courtesy of wikipedia.org. *ESC* embryonic stem cell, *iPSC* induced pluripotent stem cell

The concept of cell reprogramming is not novel (Fig. 2). It was first proposed in 1950 by Robert Briggs and Thomas King, who successfully achieved nuclear transfer of blastula cells into enucleated frog eggs [41]. In 1958, Sir John Gurdon (Nobel Prize in Medicine, 2012) cloned a frog using a technique called somatic cell nuclear transfer (SCNT). Gurdon extracted the nucleus of an intestinal cell from a *Xenopus* tadpole and injected it into a recipient enucleated frog egg [42]. The fecund egg developed into an embryo that was genetically identical to the donor. Gurdon argued that the cytoplasm of the host egg contains factors that could reprogram the genome of the differentiated cell into a totipotent one-cell-stage embryo. In 1964, a group of researchers generated PSCs from mouse embryonal carcinoma cells (ECCs) [43]. Others produced PSCs by a process of cell fusion between ECCs and somatic cells, suggesting that PSCs contain factors which confer pluripotency to somatic cells [44]. These experiments introduced the concept of “induced pluripotency” in somatic cells and extended Gurdon’s work in simple organisms, such as the tadpole, to complex mammals, and even humans. Between 1985 and 1990 different clones of PSCs were derived from human ECC lines [45–47]. A few years later, Thompson and colleagues reported the establishment of pluripotent cell lines derived from primates [48, 49] and human blastocysts [50]. In 1997, the production of the first adult cell-derived animal (a sheep known as Dolly) was achieved using the SCNT method [51]. In 2006, Shinya Yamanaka (Nobel Prize in Medicine, 2012) from Kyoto University established the first iPSCs by insertion of defined “stemness” genes into the nucleus of somatic cells [52]. These genes were retrovirally introduced into adult mouse fibroblasts and encoded four transcription factors (Oct3/4, Sox2, Klf4, and c-Myc (OSKM)) known to be involved in the maintenance of pluripotency. Yamanaka’s work transformed our understanding of epigenetic reprogramming of somatic cells to a pluripotent state and set the ground for the development of human iPSCs (hiPSCs). This can now be achieved using either the original four genes [53] or a different combination of Oct3/4, Sox2, Nanog, and Lin28 [54, 55].

### Nanog: the ever-young player in the iPSC orchestra

To date, the transcription factor Oct3/4 is thought to be indispensable for inducing pluripotency in somatic cells whereas Sox2, Klf4, and c-Myc are alternative supporting factors [56]. In 2003, Ian Chambers from the University of Edinburgh isolated a mouse gene, named Nanog, after the mythological Celtic land of the ever young, Tir nan Og. The Nanog gene is specifically expressed in PSCs and thought to be a key factor in maintaining the pluripotency state [57, 58]. Thus, it has been shown that the overexpression of Nanog in mESCs causes them to self-renew in the absence of cytokines and growth factors. Similar results were obtained with hESCs; Nanog overexpression enabled their propagation for multiple passages during which the cells remained pluripotent [59]. Conversely, the knockdown of Nanog promotes the differentiation of ESCs into other cell types, thereby demonstrating the capability of this gene to preserve the stemness state [60, 61]. Further, Nanog has been used in concert with other transcription factors to reprogram human somatic cells to iPSCs, in which it can serve as a selective marker of pluripotency [53–55, 62].

## Inducing PSCs

iPSCs are reprogrammed adult somatic cells, originally produced by retrovirus-mediated transduction of four transcription factors—Oct3/4, Sox2, Klf4, and c-Myc—known subsequently as OSKM factors [52]. The newly created iPSCs display phenotypic and functional properties of ESCs and contribute to embryonic development when injected into mouse blastocysts. Since then, mouse iPSCs (miPSCs) have been generated from embryonic fibroblasts [62], adult tail-tip fibroblasts [55], hepatocytes and gastric epithelial cells [63], pancreatic cells [64], neural stem cells [65], and B lymphocytes [66]. Additionally, researchers have reported generating iPSCs from somatic tissues of monkey [67] and rat [68]. In humans, many tissue sources have been used for successful generation of iPSCs. These include peripheral blood cells [24], cord blood cells [69, 70], keratinocytes [71], skin fibroblasts [53, 72–74], melanocytes [75], adipocytes [76], and neural stem cells [77]. Consequently, the development of hiPSCs has rapidly emerged as a promising source of PSCs, a tremendously valuable source of cells for tissue engineering, cell-based therapies, novel drug screening, as well as the molecular and cellular characterization of disease pathogenesis. Several approaches towards the generation of iPSCs have emerged. The methods used to reprogram adult cells to iPSCs can be grouped into two major categories, integrating and non-integrating methods [78].

## Integrating reprogramming methods

### Viral integration method

**Fig. 3:**
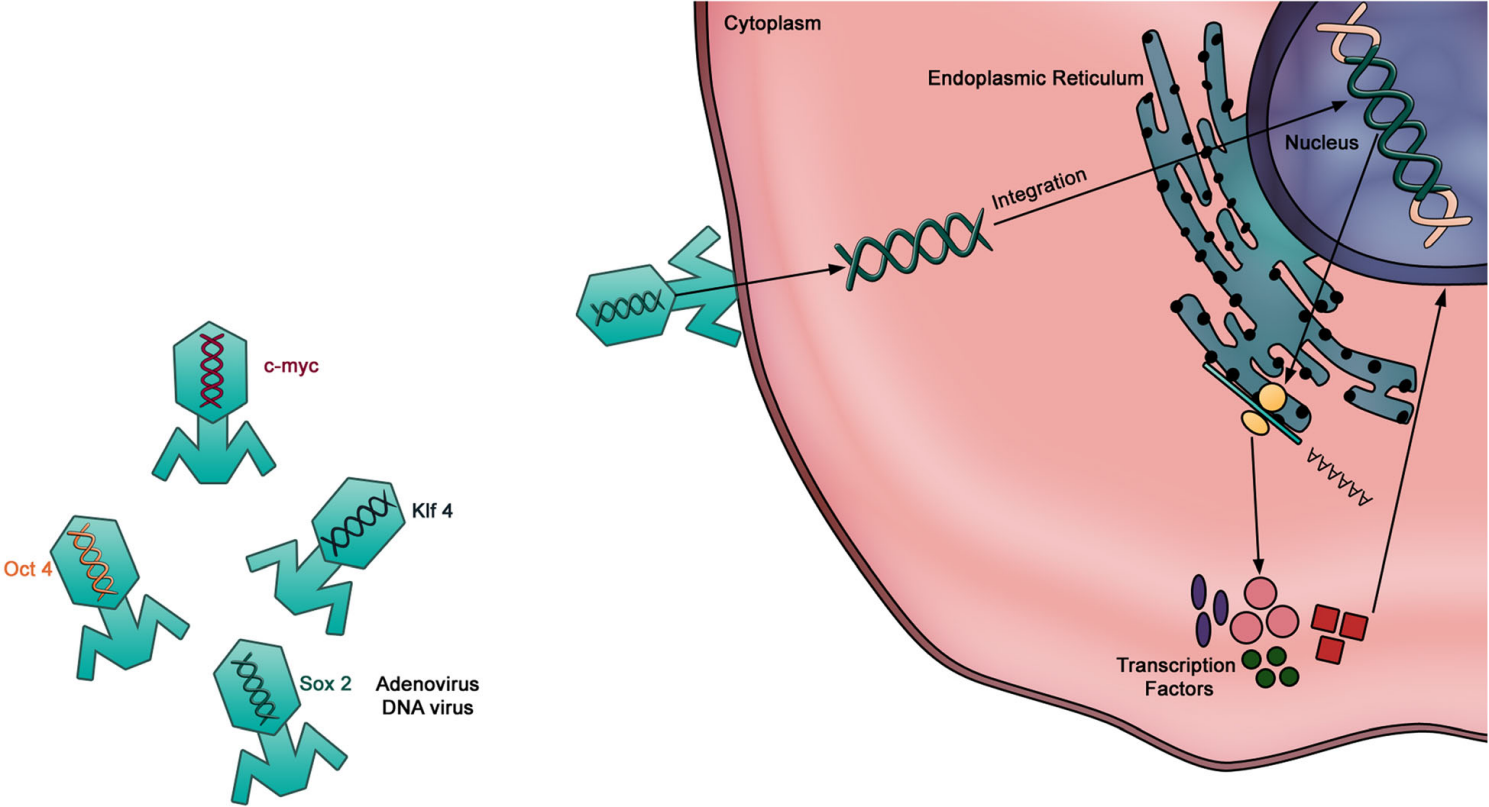
The integrating reprogramming method using viral transduction. The first method developed to deliver OSKM factors involved the use of retro- and lenti-viruses. These delivery modes were chosen based on their high efficiency. However, these methods require the reverse transcription of the delivered factors and their subsequent integration into the host genome, running the risk of induced genomic instability

The viral integration method represents the first successful approach for somatic cell reprogramming to iPSCs and uses viral delivery (retrovirus or lentivirus) of four reprogramming factors (OSKM) into the host genome [79]. In this method the transgenes carried by the viral vectors are randomly inserted into the host genome and iPSC colonies appear in culture within 3–4 weeks (Fig. 3). Expression of the transgenes is normally silenced in iPSCs, although a low level of expression or spontaneous reactivation may be observed. This may in turn affect other aspects of gene expression, DNA methylation, or pluripotency potential [72, 80–83]. As a result, such iPSCs may affect the phenotypes of their derived cells, rendering them refractory to differentiation in vitro or in vivo following transplantation. For example, c-Myc is a well-known proto-oncogene whose reactivation following retroviral gene transduction resulted in tumor formation in almost 50% of chimeric mice generated from iPSCs [62, 84, 85]. Therefore, other reprogramming factors have been screened and c-Myc-free iPSCs were generated using a combination of four or three of the Oct3/4, Sox2, Nanog, and Lin28 factors [54, 55, 85–87]. These alternative approaches were successful in the production of iPSCs without transgenic insertion of c-Myc, albeit with reduced efficiency [55, 84]. Other studies have further reduced the number of genes required for reprogramming to one or two factors using Oct3/4 alone [77, 88] or in combination with Sox2 or Klf4 [65, 89–91]. Of note, the omission of one or more of the reprogramming factors is largely dependent on the endogenous expression of these factors in the donor cell type. For example, hiPSC derivation using the lentiviral system takes several weeks with skin fibroblasts but only 10 days with keratinocytes, in which the expression levels of Klf4 and c-Myc are much higher [92]. Therefore, the best combination of reprogramming factors is partly dependent on the hosting cell type.

### Viral integration followed by excision: the Cre-Lox system

**Fig. 4:**
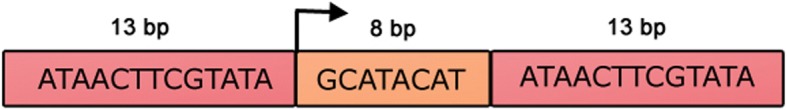
Lox site. The 8-bp core sequence is flanked by two 13-bp inverted repeats

**Fig. 5:**
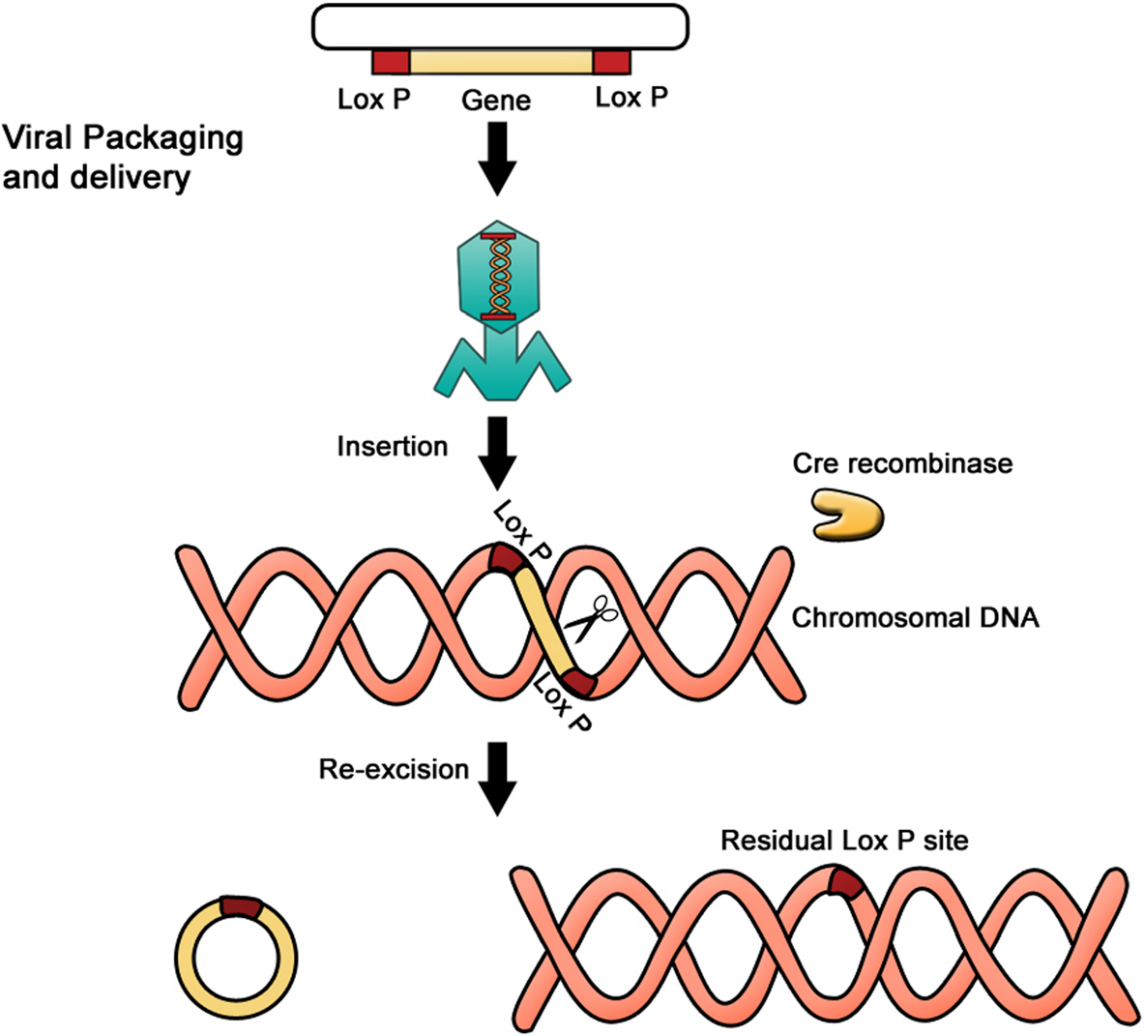
The Cre-Lox excision system. The DNA sequences for the OSKM factors are flanked by LoxP sites and delivered virally to the target cells of interest. The Cre-recombinase is delivered in parallel in a similar manner. When expressed, the Cre-recombinase excises the sequences by recombination of the two flanking LoxP sites. This excision will nevertheless leave a residual LoxP site at the site of the original insertion

The problem of permanent integration of transgenes in a host genome was partially solved by viral integration of OSKM factors into the host genome followed by their excision using the Cre-Lox recombinase system (Fig. 4). In mammalian cells, Cre-Lox recombination is widely used to control gene expression, induce chromosomal rearrangement, or delete undesired DNA segments (Fig. 5) [93, 94]. In the context of hiPSCs, LoxP-lentiviral vectors containing either four (Oct3/4, Sox2, Klf4, c-Myc) or three (Oct3/4, Sox2, Klf4) reprogramming factors flanked between two unidirectional LoxP sites have been employed [95]. The hiPSCs are then transiently transfected with an expression vector encoding Cre-recombinase that mediates the excision of the integrated transgene (Fig. 5). This has the advantage of inducing the generation of transgene-free hiPSCs, favoring the translation of iPSC technology into clinical applications. Despite the efficiency of Cre-recombinase-driven excision and the advantages of this approach, residual viral vector sequences can remain at the sites of integration, which may in turn trigger undesirable downstream effects, while the overall reported reprogramming efficiency remains very low.

### Non-viral integration followed by removal: the PiggyBac transposition

**Fig. 6:**
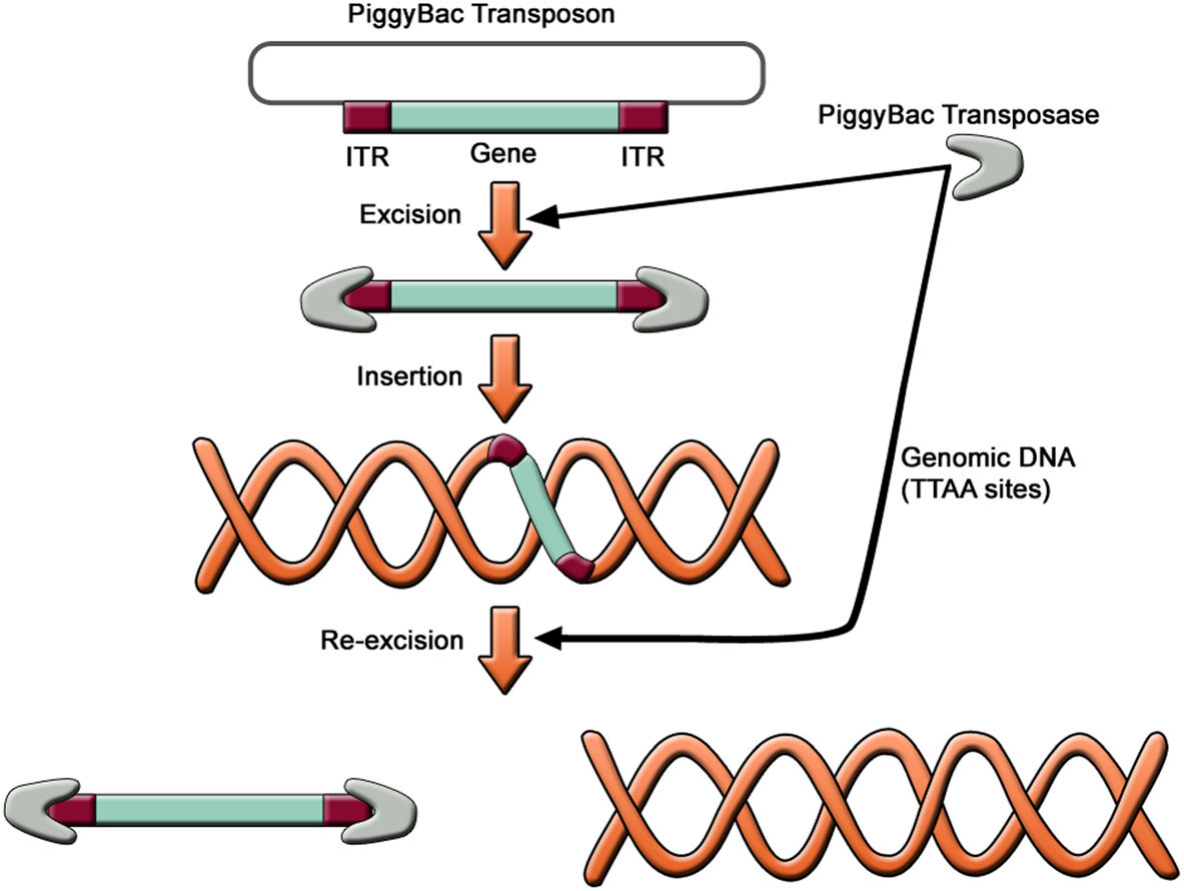
The PiggyBac transposition system. The PiggyBac transposase has the ability to integrate into the genomic DNA of the host cell a DNA sequence provided that it is flanked by ITR sequences. The same PiggyBac transposase can in turn excise this inserted material, leaving the genomic DNA virally unchanged. *ITR* inverted terminal repeat

In order to avoid viral integration altogether, transposon-based non-viral integration methods have been developed using the PiggyBac (PB) transposon system. The PB transposons are mobile genetic elements used to transpose target sequences between vectors and chromosomal DNA via a “cut and paste” mechanism (Fig. 6) [96]. The procedure consists of co-transfecting cells with PB transposon vectors (containing target sequence) and PB transposase expression plasmids. The PB transposase recognizes specific inverted terminal repeat (ITR) sequences located on both ends of the transposon vector, efficiently removes the contents from the transposon sites, and integrates them into TTAA chromosomal sites. Cells harboring an inserted PB vector are transiently re-transfected with the PB transposase expression vector. The PB transposase substantially re-excises the transposons from the genome, “footprint”-free.

Transgene-free iPSC lines were generated from human embryonic fibroblasts (hEFs), human embryonic kidney 293 (HEK293) cells, and adult skin fibroblasts using the PB transposon-based system [97]. This approach has several advantages over the traditional viral integrating methods for reprogramming. First, the plasmid DNA and the transfection protocol used for cell delivery of PB transposon vectors are innocuous and offer the opportunity to reprogram cell types that are prone to viral infection. Second, the feasibility of the protocol and the reliability of the PB transposase-mediated excision enhance the establishment of transgene-free hiPSC lines. However, this approach results in low yields (< 2%) of bona fide iPSCs. Of note, it has been shown that the efficiency of iPSC derivation from human adult fibroblasts using PB transposon vectors is enhanced by 15- to 51-fold after addition of butyrate, a small-chain fatty acid [98]. The mechanism of butyrate action includes histone acetylation, DNA demethylation, and the expression of endogenous pluripotency associated genes.

Although remarkable progress has been made towards safe and efficient reprogramming, the aforementioned methods involve integration of transgenes into the host genome with unpredictable interruptions to the host cell genome and downstream consequences. In order to avoid any permanent or transient genomic modifications a safer approach for iPSC derivation is to avoid both permanent and transient genomic modification. Therefore, non-integrating methods for cell reprogramming have been developed and considered.

## Non-integrating reprogramming methods

### Viral non-integrating method

**Fig. 7:**
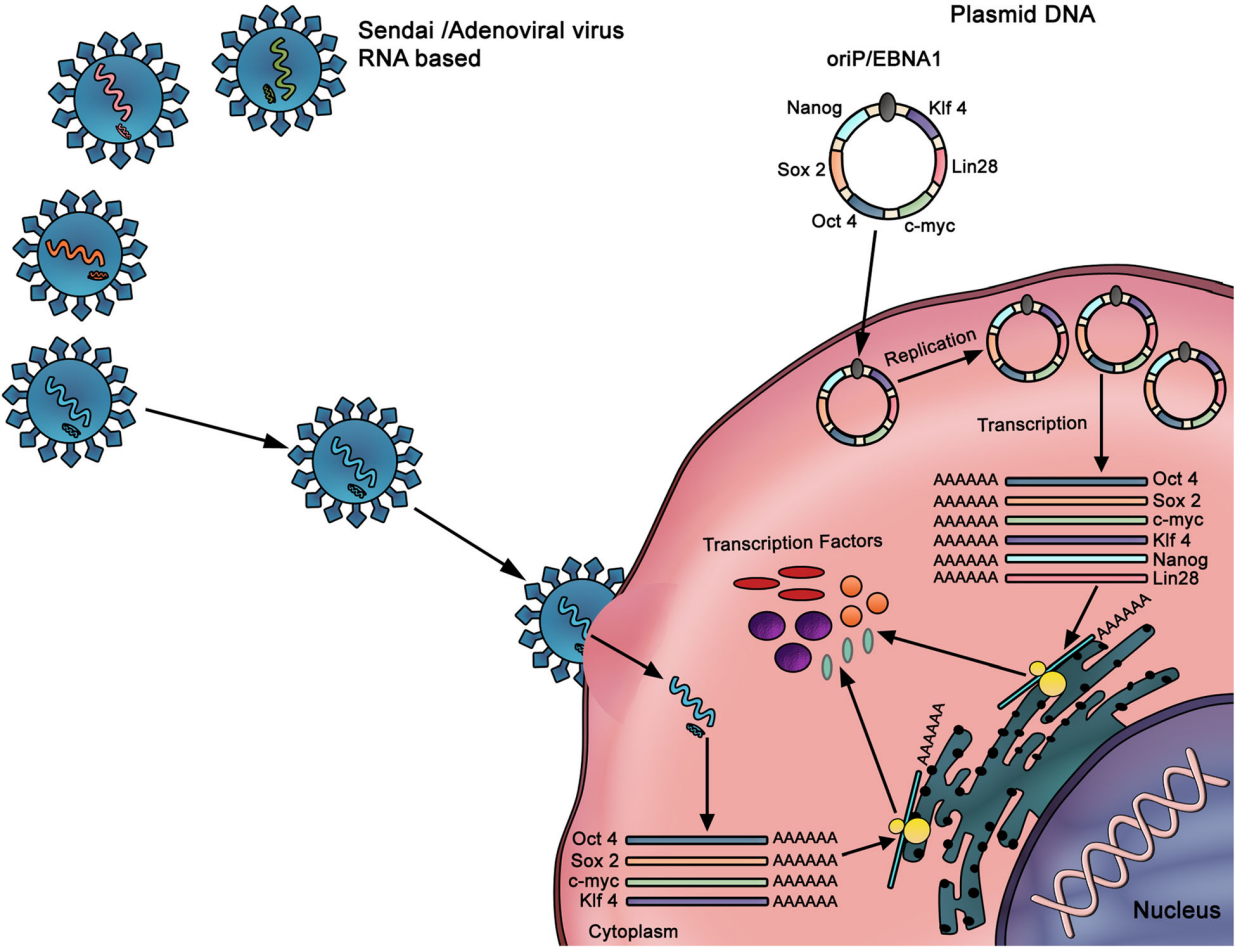
Non-integrative methods using plasmids, sendaiviruses, or RNA delivery. Non-integrative methods (DNA- or RNA-based) have been developed to overcome the increased risk of genomic instability and gene expression modifications encountered with integrative methods. When RNA-based, the mRNA is delivered without reverse transcriptase and is directly translated into proteins. The RNA can be delivered directly or using viruses. The DNA can also be directly delivered to the target cells in a form of self-replicating plasmid that will not integrate the host cell genome. The plasmid is then transcribed to mRNA for translation to proteins. *O* Oct3/4, *S* Sox2, *K* Klf4, *M* c-Myc

The viral non-integrating method involves the generation of iPSCs using non-integrating viruses such as adenoviruses and sendai viruses for the delivery of OSKM factors (Fig. 7). As opposed to retroviruses and lentiviruses, these expression vectors do not integrate into the host genome and show high-level expression of exogenous genes [99–101]. So far, the adenoviral/sendaiviral iPSCs display features of reprogrammed cells, express endogenous pluripotency genes, and contribute to tissue development in chimeric mice. Furthermore, viral genome and viral proteins were totally absent in iPSC clones generated by adenoviral or sendaiviral transduction. However, major issues are hindering the long-term success of this method. For example, in most cases, iPSC lines generated by adenoviral/sendiviral transduction formed teratomas when injected into immunodeficient mice [99–101]. Furthermore, Stadtfeld and colleagues found that almost 25% of the adenoviral iPSC lines were tetraploid, which is not seen in iPSCs produced with retro- or lentiviral vectors [99]. The authors postulate that adenoviral reprogramming either induces cell fusion or, alternatively, selects for rare tetraploid cells pre-existing in the starting cell populations. In addition, the efficiency of deriving iPSCs was ~ 100-fold lower than that obtained with integrating viruses. This is probably due to the fact that many cells do not maintain gene expression of OSKM factors long enough to trigger entry into a pluripotent state.

### Non-viral non-integrating methods

Non-viral non-integrating methods consist of the derivation of iPSCs through virus-free and transgene-free techniques. This relies on the induction of iPSCs by transient transfection of plasmid DNA, minicircle DNA, or synthetic RNA encoding OSKM factors, as well as the direct delivery of recombinant proteins of OSKM factors into the cells.

#### Plasmid DNA

When transfected into cells, plasmid DNA replicates independently of the genomic DNA without incorporating into the genome of the host cells. Transgene-free iPSCs have been produced from mouse [102] and human [100, 103] fibroblasts by transient transfection with plasmid vectors. In particular, hiPSCs were generated by repeated transient transfection with three plasmids expressing seven reprogramming factors. These factors include Oct3/4, Sox2, c-Myc, Klf4, Nanog, and Lin 28, along with Epstein-Barr nuclear antigen-1 (EBNA-1), and SV40 large T antigen (SVLT), which allow stable extra-chromosomal replication of the plasmid vectors [100]. Interestingly, the omission of the later factor resulted in cell toxicity and disappearance of iPSC colonies. Although the isolated hiPSCs were devoid of vector or transgene expression, the differentiation process remained extremely low and required repetitive transfections.

#### Minicircle DNA

Minicircle DNA are small supercoiled derivatives of plasmids that are free of all prokaryotic vector sequences and are composed essentially of a small eukaryotic expression cassette (~ 4 kb). The absence of bacterial DNA backbone makes them powerful tools for genetic manipulation of mammalian cells. In addition, their small size enhances their transfection capacity and confers a long ectopic expression pattern compared to standard plasmids [104, 105]. Minicircle vectors carrying a cassette of the transcription factors Oct3/4, Sox2, Lin28, and Nanog have been employed for derivation of hiPSCs from adipose stromal cells [106] and neonatal fibroblasts [107]. No genomic integration of the minicircle transgene has been detected in hiPSC subclones as confirmed by Southern blot analysis. However, the reprogramming efficiency remains extremely low (0.0005–0.005%) compared to viral integration techniques used for the expression of the same transcription factors [54, 55].

#### RNA delivery

The RNA-based method for somatic cell reprogramming consists of delivering OSKM factors by repeated administration of synthetic messenger RNA (mRNA), an approach that overcomes viral genome integration or immune responses to foreign DNA. Multiple human cell types have been reprogrammed using synthetic modified messenger RNA [108]. Furthermore, the same technology has been employed to differentiate the mRNA-induced iPSCs into myogenic cells. Recently, the use of selected microRNAs (miRNAs) with or without OSKM factors has been shown to be an efficient method of producing iPSCs [109–111]. The mechanism by which miRNAs enhance iPSCs reprogramming is unclear, but it could be related to their ability to regulate the cell cycle [111]. Of note, several miRNAs used in the reprogramming process are usually expressed in ESCs and are thought to maintain the ESC phenotype [112, 113]. The RNA-based method represents a promising strategy to reprogram somatic cells with less or no genetic modifications, qualifying mRNA-reprogrammed cells for clinical applications. Nonetheless, this approach entails a small risk of genetic modification due to the introduction of nucleic acids into the cell.

#### Protein delivery

**Fig. 8:**
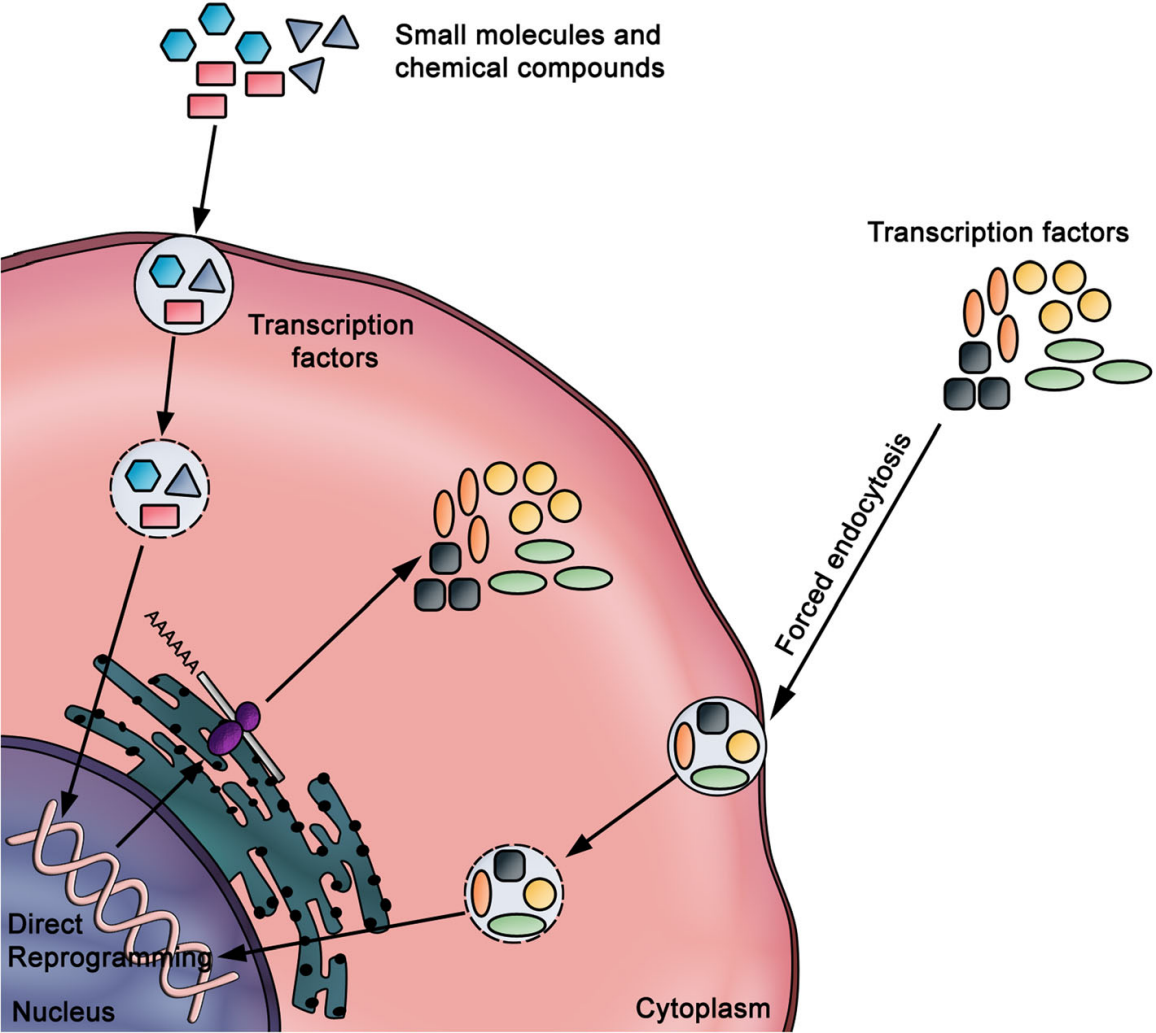
Direct reprogramming using transcription factors or small molecules. To avoid the use of genetic material, fibroblasts can also be reprogrammed by the excessive delivery of OSKM factors in their protein form. The method consists of the incubation of fibroblasts with a large amount of OSKM factors and their internalization by forced endocytosis. The factors then bind to DNA and directly induce the reprogramming of the target cells. The use of small molecules and chemical compounds during the reprogramming process could significantly improve the efficiency of the reprogramming process

The protein delivery method involves the direct delivery of reprogramming factors (i.e., proteins) into the cell (Fig. 8). Through this approach, hiPSCs have been successfully generated from mouse [114] and human neonatal fibroblasts [115] by direct delivery of the OSKM factors conjugated with a cell-penetrating polyarginine peptide. Of note, this method has an attractive advantage of being virus-free and does not include genetic modification or DNA transfection. However, the low reprogramming efficiency and the need for repeated treatments represent the major limitations.

## Improving iPSC reprogramming efficiency

Numerous chemicals and small molecules have been shown to improve the efficiency of iPSC generation or enable the reduction of the reprogramming factors required for pluripotency induction [116]. These molecules and compounds can be divided into two groups: 1) chromatin modifiers and 2) regulators of cell signaling pathway [117]. For instance, valproic acid (VPA) is a small molecule histone deacetylase inhibitor which has been used to successfully reprogram foreskin fibroblasts with only two factors: Oct3/4 and Sox2 [89]. The reprogramming efficiency was significantly improved when VPA was applied to cells expressing high endogenous levels of c-Myc and Klf4, such as keratinocytes or adipose stromal cells [92, 118]. Other studies optimized the reprogramming efficiency by combining two or three small molecules with transcription factors. For example, neonatal epidermal fibroblasts have been reprogrammed by using Oct3/4 and Klf4 supplemented with CHIR99021 (Wnt signaling pathway activator) and Parnate (histone demethylase inhibitor) [119]. Similarly, the combination of SB431542 (transforming growth factor, TGF-β inhibitor), PD0325901 (MEK inhibitor), and thiazovinin (cell-survival enhancer) significantly promotes the reprogramming efficiency of fibroblasts [119]. Also, the addition of vitamin C together with VPA to serum-containing culture media improved reprogramming efficiency by threefold compared with VPA alone [120]. Despite the tremendous efforts invested to achieve a high reprogramming efficiency, the yields of bona fide hiPSCs have rarely exceeded 1%. Two conflicting models have been proposed to explain the renitence to pluripotency induction, namely the “elite” and “stochastic” models [121, 122]. The elite model postulates that only a small fraction of somatic cells, most likely the tissue-resident stem cells, are subjected to reprogramming. The stochastic model argues that under specific culture conditions, either tissue-resident stem cells or fully differentiated cells can be successfully reprogrammed to a pluripotent state in a stochastic fashion [64, 66, 123]. Further investigation is needed to establish a consensus model that allows a better understanding of the mechanisms of reprogramming at the multicellular and single-cell levels.

## Characterization of iPSC lines

Reprogramming of somatic cells is hindered by the heterogeneity of the derived iPSC lines, which affects their differentiation potential into specific cell lineages. Even a single reprogramming experiment could generate multiple iPSC lines which exhibit distinct molecular and functional characteristics [124–126]. This problem is largely due to the differential propensity to pluripotency induction among cells and our limited understanding of the underlying reprogramming mechanisms. In this context, several methods have been employed to evaluate the characteristics of established iPSC clones. Whole genome expression or quantitative reverse-transcription polymerase chain reaction (qRT-PCR) can be used to assess the gene expression signatures of the iPSC clones, while immunocytochemistry and western blots are employed to examine protein expression. The differentiation potential of iPSC clones can be assessed in vitro by embryoid body formation and in vivo by teratoma formation after transplantation in animals. In another exciting approach, Chan and colleagues attempted to define the molecular signature of the fully reprogrammed hiPSCs using in situ live cell imaging [127]. They found that transgene silencing and expression of the pluripotency markers TRA-1-60, DNA (cytosine-5-)-methyltransferase 3 beta **(**DNMT3B), and REX1 marked the fully reprogrammed state whilst alkaline phosphatase, SSEA-4, growth differentiation factor 3 (GDF3), human telomerase reverse transcriptase (hTERT), and Nanog are insufficient as markers. Recently, Burridge and colleagues claimed to have established culture conditions that circumvent the interline variability of iPSC lines, which could significantly facilitate the downstream characterization of the reprogrammed iPSCs and increase the number of suitable iPSCs for the needs of each project [128].

## Host cells used for iPSC reprogramming

### Fibroblasts

The vast majority of studies on hiPSC derivation from somatic cells have employed dermal fibroblasts as the starting population for reprogramming [129–131]. Fibroblasts play an important role within the dermis and are responsible for the synthesis of connective tissues and remodeling of the extracellular matrix. They can be obtained from a single skin biopsy followed by 3–4 weeks of in vitro incubation to generate a sufficient amount of starting cell population [132]. Their easy isolation and expansion renders them the best source of iPSCs. However, the efficiency of reprogramming is very low, ranging from 0.0001% (when using reprogramming factors without c-Myc) to 0.01% (in the presence of c-Myc) [53, 55, 89, 132]. In addition, the time required for the formation of iPSCs is relatively long and colonies usually take up to 2 months to appear in culture [133]. However, recent reports suggest approaches that increase efficiency of reprogramming of primary fibroblasts [129, 130].

### Keratinocytes

Keratinocytes, the most abundant cell type in the epidermis, are involved in the protection of the skin and provide strength to the hair and nails. One study has reported the generation of iPSCs from keratinocytes obtained from human foreskin biopsies and plucked hair [71]. These cells showed a significant improvement in reprogramming efficiency and speed compared to skin fibroblasts. However, the keratinocytes used in this study were derived from neonatal and juvenile individuals. In yet another study, iPSCs were established from human hair follicle keratinocytes, suggesting that some microenvironmental cues of hair follicles may allow for efficient hair follicle re-differentiation [134]. Recently, integration-free iPSCs have also been established from keratinocytes of healthy donors [135].

### Melanocytes

Melanocytes are skin-specialized cells responsible for the production of melanin, the darkening pigment of the skin. Similar to fibroblasts and keratinocytes, melanocytes have been derived from skin biopsies and expanded in vitro [75]. When compared to fibroblasts, these cells showed a higher reprogramming efficiency and speed using the four OSKM factors. Interestingly, melanocytes express high endogenous levels of Sox2 and can be reprogrammed with only three factors (Oct3/4, Klf4, and c-Myc). Unfortunately, the age of the melanocyte donor was not indicated in this study, thus limiting the comparison with other cell types. More recently, a new protocol for deriving iPSCs from melanocytes in serum-free culture has been described [136], making their application in regenerative medicine potentially more feasible.

### Fetal neural stem cells

The major advantage of fetal neural stem cells is their ability to be reprogrammed using only the Oct3/4 factor [77]. However, their fetal origin makes the comparison to other cell types difficult, while the invasive procedures required for their isolation limits their potential usage.

### Cord blood cells

Cord blood cells (CBCs) have also been used to derive iPSCs. In fact, CD133^+^ cells isolated from freshly isolated or cryopreserved cord blood units have been reprogrammed to iPSCs using Oct3/4 and Sox2 [69]. Another study has reported the generation of iPSCs from cord blood-derived endothelial cells using Oct3/4, Sox2, Nanog, and Lin28 [70]. CBCs can be readily collected from the umbilical cord at birth without invasive procedures. Unlike ASCs, CBCs are neonatal stem cells which have a reduced risk of acquiring and transmitting somatic mutations onto the derived iPSCs and retain the immunological immaturity of neonatal cells. However, CBCs comprise different populations of cells, including HSCs [137], mesenchymal stem cells [19], and endothelial progenitor cells [138]. This mixing of cells could generate a heterogeneous population of derived iPSCs with low reprogramming efficiency [69, 70]. Of note, patient-specific CBC-derived iPSCs would be available for patients who had their cord blood banked at childbirth. Thus, the long cryopreservation time may alter the reprogramming efficiency and the regenerative therapy of these cells.

### Peripheral blood CD34^+^ cells

CD34^+^ cells are a subset of stem cells with a therapeutic potential against multiple hematologic malignancies and immunodeficiency disorders. Cells expressing CD34^+^ are normally found in the bone marrow; however, the administration of some cytokines, such as the granulocyte colony-stimulating factor (G-CSF) and the granulocyte-macrophage colony-stimulating factor (GM-CSF) enhance their trafficking to the peripheral blood [139]. This process, known as stem cell mobilization, can markedly increase the number of circulating CD34^+^ to ~ 1% of the total cell count, offering an abundant source of progenitor cells for reprogramming [140]. Peripheral blood CD34^+^ cells have been used as a starting population for iPSC derivation using the OSKM factors [24]. So far, the reprogramming efficiency of these cells is comparable to skin fibroblasts. However, in vitro expansion of CD34^+^ cells is challenging. Furthermore, the intake of G-CSF for mobilization may lead to undesirable effects, especially in patients with cardiovascular diseases such as headache, nausea, and bone pain [141].

### Adipose-derived stem cells

Adipose tissue is a specialized connective tissue derived from embryonic mesenchyme that contains a mixture of multipotent stem cells that have the potential to differentiate into multiple cell lineages, including bone, cartilage, and muscle [26, 142, 143]. Adipose-derived stem cells (ADSCs) are derived by aspiration of adipose tissue (lipoaspiration) and can be directly reprogrammed to iPSCs using the four OSKM factors [76]. A high amount of ADSCs could be collected from a small amount of lipoaspirates following a short culture period (~ 48 h). In addition, the reprogramming of ADSCs does not require the support of mouse feeder cells for the reprogramming, thereby avoiding the possibility of contaminating the derived iPSCs with animal pathogens. In comparison with human fibroblasts, the reprogramming efficiency of human ADSCs was 20-fold higher and twofold faster and the expression levels of Klf4 and c-Myc are relatively high [76, 118]. The abundance of adipose tissue, the ease of harvesting of ADSCs, the pluripotency capacity, and the low morbidity put ADSCs at the top of the somatic cell list for use in reprogramming.

## Saving the failing heart: iPSC differentiation into cardiomyocytes

In spite of promising pharmacological and surgical interventions in CVD, heart transplantation remains the sole therapeutic option for end-stage heart failure. Alternative approaches may include the refurbishment of the CM population to rescue the failing myocardium and restore heart function. The derivation of CMs from hiPSCs is a novel therapeutic strategy that could transform the future of cardiovascular medicine. However, the establishment of differentiated CMs that fulfill this purpose requires a substantial improvement of hiPSC culture methods and CM differentiation. Various methods have been described to induce the differentiation of iPSCs into CMs. These methods are closely related to those traditionally employed for the derivation of CMs from hESCs, since hiPSCs and hESCs share similar characteristics and differentiation potential.

### Small-scale protocols of differentiation

**Fig. 9:**
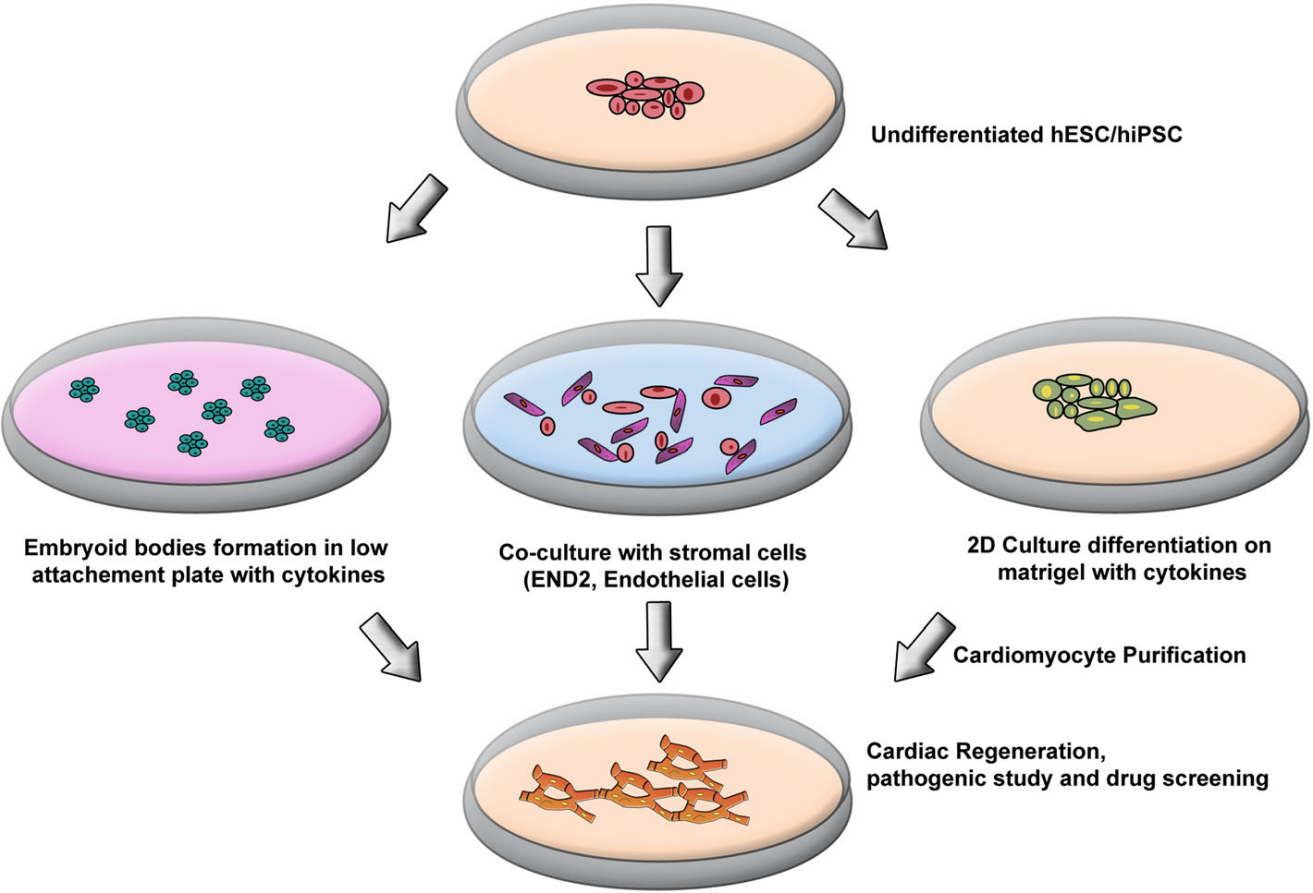
In vitro differentiation of CMs from hiPSCs. Three main methods are documented for differentiation of hiPSCs into CMs. The most documented, directed cardiac differentiation, is achieved with sequential cytokine stimulation following the culture of hiPSCs in low adherent culture plates, forcing the cells to aggregate into so-called embryoid bodies. Alternatively, the same type of sequential cytokine stimulation was also proven successful when cells are kept in 2D conditions. Finally, a “natural” differentiation into CMs was documented following co-culture of hiPSCs with END-2 endothelial cells. *CM* cardiomyocyte, *END-2* endodermal cell line-2, *hiPSC* human induced pluripotent stem cell

In general, three small-scale PSC-to-CM differentiation strategies have been implemented: 1) embryoid body (EB) formation assays; 2) co-culture of undifferentiated PSCs with a visceral endodermal cell line (END-2); and 3) a confluent PSC monolayer in the presence of defined cardiogenic growth factors (Fig. 9).

#### Embryoid body formation assays

EB formation assays are the most common method to generate CMs from iPSCs. EB assays involve the growth of undifferentiated iPSCs as aggregates in suspension, causing them to form structures called EBs [144–147]. Formation of EBs has been reported with different approaches, including static suspension culture, hanging drops, and forced aggregation, followed by stage-specific application of cardiogenic factors. Under serum-free conditions that do not support pluripotency and with the supplementation of several cytokines, such as activin A and BMP4, EBs can efficiently differentiate into beating CMs [147–152]. Zhang and colleagues reported the derivation of functional CMs from the EBs of hiPSCs that were lentivirally transduced with Oct3/4, Sox2, Nanog, and Lin28 [153]. These hiPSC/EB-derived CMs were comparable to those generated from hESCs and expressed similar phenotypical, structural, and functional characteristics. More specifically, cultures of hiPSC-derived CMs have shown down-regulation of Oct3/4 and Nanog as well as upregulation of cardiac genes, contractile protein expression, and sarcomeric organization. Moreover, the cells generated atrial, nodal, and ventricular action potentials (APs) and responded adequately to electrical stimulation and pharmacological activation of the β-adrenergic signaling pathway. The authors noted that the contractility of hiPSC-CMs was less than that of hESC-CMs and the silencing of the transgenes Oct3/4 and Nanog was not as efficient. However, these differences normally occur among cell lines derived from the same PSC population and are shared between all differentiation methods.

#### Co-culture of PSCs with visceral endoderm-like cells

Visceral endoderm is an extraembryonic cell layer formed in the early stage of embryonic development that secretes critical factors involved in embryonic development. Mummery and colleagues reported that co-culture of human and mouse PSCs with visceral END-2, derived from mouse P19 embryonal carcinoma cells, can efficiently induce their differentiation into CMs [150, 154, 155]. Although the cardio-inductive mechanism of END-2 is unclear, the transcriptome and secretome profiles have been determined [156, 157]. Analysis of the serum-free media conditioned by END-2 revealed that SB203580, a specific p38 MAP kinase inhibitor, and prostaglandin E are potent promoters of cardiac differentiation [158], whereas insulin or insulin growth-factor-1, activators of the PI3/Akt signaling pathway, act as potent inhibitors [158, 159].

#### Confluent PSC monolayer differentiation by specific cardiogenic growth factors

This method consists of direct differentiation of iPSCs towards the cardiac lineage by sequential addition of defined growth factors known to induce cardiac development in various animal models. This sequential addition of specific growth factors aims to recapitulate, in vitro, the embryonic development of heart tissue. Nodal signaling in the ectoderm evokes mesoderm induction, thus marking the onset of gastrulation. The role of Nodal in the development of germ layers and the primitive streak is crucial. Indeed, loss of Nodal function has been shown to lead to loss of mesoderm and excessive ectoderm, as well as embryonic lethality during early gastrulation [160, 161]. When gastrulation ensues, mesodermal cells start to emerge from the primitive streak. Among the earliest cell lineages to emerge are cardiac progenitor cells. These cells express a myriad of mesoderm genes, including Wnt3, Brachyury T, BMP4, and MESP-1 [162–164]. As a major determinant of cardiovascular lineage commitment, MESP-1 orchestrates the increased expression of several transcription factors involved in cardiac differentiation and maturation, such as GATA4, NKx2.5, Mef2c, and Tbx5 [165, 166]. Moreover, by virtue of its ability to directly inhibit Wnt and Nodal via DKK1 and CER1, MESP-1 imparts a strong repressing effect on early mesoderm induction [167, 168]. Based on the above, approaches that stimulate human PSCs with successive rounds of recombinant growth factors such as basic fibroblast growth factor (bFGF), BMP4, Wnt3, and Activin A, followed by addition of DKK1 or other Wnt inhibitors, have been employed to induce cardiac differentiation [148, 149, 169]. In addition, other modulators such as Noggin [170], VEGF [148], CHIR and IWR-1/IWP-2 [171, 172], TGF-β signaling inhibitor [173], and SHH signaling activation [173] have been shown to increase the differentiation efficiency.

### Large-scale protocols

Although small-scale protocols are successful in producing a high percentage of iPSC-derived CM, they suffer from limited scalability, limited reproducibility, and heterogeneity. Moreover, large animal models, high-throughput assays, and tissue engineering need a constant supply of billions of CMs, which require, more advanced and scalable strategies. Large-scale production using 2D culture was successfully achieved by scaling out the culture surfaces. This approach, however, is not cost- or space-efficient. Therefore, mass production of PSC-derived CMs was successfully implemented using 3D industry-compatible platforms. Such platforms include matrix-dependent cultures, such as microcarrier suspension cultures and sphere culture with gellan gum polymer, and matrix-independent suspension cultures, including spinner flasks and bioreactors. Transition to 3D cultures require the generation of suspension aggregates from dissociated clumps, microcarriers, self-assembling aggregates, or forced aggregation by micropatterning. Maintenance of aggregates in homogenous conditions is achieved by rocking, agitating, or stirring the culture depending on the platform format being used. Multiple studies have successfully produced high yields of ventricular-like CMs in a large scale and from different hPSC lines using multiple chemical modulators and different bioreactors [172, 173]. Excellent reviews describing large-scale techniques in detail can be found elsewhere [174, 175].

### Improving CM differentiation

Several approaches geared towards improving the differentiation and maturation of iPSC-derived CMs have been suggested. Some of these promising strategies include knockdown of certain genes [176], bioreactors [177], hypoxic culture conditions [178–181], controlled feeding strategies and variation in chemical supplementation [172, 173], as well as aggregation of iPSC-derived EBs in chemically pre-defined medium [126]. Combining hypoxia and bioreactor hydrodynamics to boost iPSC differentiation into CMs has been established [177]. Correia and colleagues explored the impact of dissolved oxygen (DO) at 4% tension and mechanical forces using two distinct bioreactor systems, namely WAVE (high mechanical loading frequency) and stirred tank (low mechanical loading frequency) bioreactors [177]. They found that intermittent agitation with changes of stirring direction in stirred bioreactors led to high cell lysis and low CM numbers, but higher yields when compared to normoxic conditions (20% O_2_ tension) [177]. This is in line with other bioengineering technologies that are geared to transform the discipline of regenerative medicine [182].

With WAVE bioreactors, however, wave-induced agitations and high mechanical loading led to sixfold lower increase in cumulative lactate dehydrogenase (LDH) with higher CM yields and faster kinetics compared to stirred tanks. Additionally, 97% CM purity by puromycin selection was achieved in 2 days (total 11 days of differentiation) with WAVE bioreactors versus 7 days (total 16 days of differentiation) with stirred tank cultures [177]. These findings are interesting since it is been shown that CMs isolated at day 11 of differentiation survived cardiac engraftment following intramyocardial transplantation better compared to CMs differentiated for 16–18 days [183]. Ting and colleagues also demonstrated that an intermittent rocking platform (Wave type) to integrate micro-carrier suspension resulted in much higher CM yields than stirring platforms, which showed reduced CM yields compared to static microcarrier cultures [184].

Another strategy consists of replacing the mouse embryo fibroblast (MEFs) feeder layer with human cells. Current methods of hiPSC culture involve the utilization of a feeder layer of MEFs. These inactivated MEFs are known to promote the proliferation of hiPSCs as well as to maintain them in an undifferentiated state. This, however, is not without the risk of exposing the cultured hiPSCs to animal contaminants. Attractively, however, and as has been published with hESCs, MEFs can be efficiently replaced by culturing autologous skin fibroblasts obtained from the same donor/patient [185, 186]. Alternatively, matrigel-coated surfaces have also been utilized with promising results [187]. Application of a layer of synthetic matrices over the monolayer culture (sandwich method) in addition to the sequential application of growth factors further promotes hPSC-CM differentiation [188]. Burridge and colleagues developed an optimized cardiac differentiation that produced contractile sheets of up to 95% troponin-positive cardiomyocytes in 11 hiPSC lines. Their strategy was based on using synthetic matrices and a chemically defined medium consisting mainly of RPMI 1640, L-ascorbic acid 2-phosphate, and rice-derived recombinant human albumin along with other small molecules [189].

In an attempt to define the various molecules that could promote differentiation of iPSCs to CMs, a high-throughput screening system has been developed. Some of the identified molecules include resveratrol [190], vitamin C [120], cyclosporine A [191], and triiodothryonine [192]. Moreover, it was reported that differentiation and maturation of hESCs and hiPSCs may be potentiated by activation of Wnt/β-catenin signaling [193] or by exogenously expressing human apolipoprotein-A1 [194]. These cardiogenic effects are thought to be mediated by the BMP4/SMAD signaling pathway. Of note, manipulation of differentiation protocols using different protein factor concentrations and treatment strategies, matrix components, or SMs resulted in large variations in CM differentiation efficacy among different cell types and lines, suggesting the importance of optimization procedures [173, 174, 195, 196]. In addition to different protocols, a key player that influences the differentiation potential is the cellular origin of iPSCs [197]. This is not surprising given the notion of “epigenetic memory” of iPSCs, which dictates various aspects of gene expression and differentiation potential [197–199]. iPSCs derived from cardiac lineage cells are believed to be more effective for transplantation and engraftment than non-cardiac lineage-derived iPSCs [190, 200, 201]. Sanchez-Freire and colleagues compared the effect of human donor cell source on CM differentiation and function of derived iPSCs [200]. They found that human cardiac progenitor cells (CPCs) have higher CM differentiation efficiency than human skin fibroblasts of the same donor due to epigenetic differences. However, iPSC-CMs derived from both cell types have similar therapeutic capabilities after implantation in an animal MI model [200]. Chun and colleagues studied the impact of different types of anisotropic mechanical strain on iPSC-CMs derived from skin fibroblasts of healthy versus dilated cardiomyopathy (DCM) patients [202]. They revealed that genetic backgrounds carried from healthy and DCM patients highly influence responses to different types of strain conditions [202].

In summary, many factors play critical roles in influencing the differentiation of iPSCs to CMs. Some of these include the starting cell population, cardio-inductive molecules and growth factors, as well as culturing conditions. Empirically determined optimum employment of these factors is key for successful and efficient differentiation of iPSCs to CMs.

## Purification and enrichment of iPSC-derived CMs

Subsequent to differentiation, CMs need to be purified and enriched. To this end, several commonly methods are employed. These include the use of a pulled-glass micropipette for manual separation [151], density gradient-based separation [203], fluorescence-activated cell sorting (FACS) [204], metabolic purification [205], as well as antibiotic selection [206]. While manual dissection/separation or density gradient-based separation show limited success at enrichment, antibiotic selection yields significantly higher CM purity [177]. The use of FACS is due to the ability of this technique to provide a positive selection of CMs that are phenotypically different from other cells. To this end, a set of surface proteins can be used as markers for the enrichment of CMs. These include CD166 [207], vascular endothelial growth factor receptor 2 (VEGFR2) and platelet-derived growth factor receptor-α (PDGFR-α) [208], elastin microfibril interface 2 (EMILIN2) [209], signal regulatory protein-α (SIRPA-α) [210], and vascular cell adhesion protein1 (VCAM1) [210, 211]. A major limitation for this approach is the lack of specific CM surface markers that could identify and select cardiac progenitor cells from a pool of differentiating/undifferentiating cells [212]. To overcome this problem, genetically modified hESC lines that allow for selection of terminally differentiated CMs have been developed. This approach is based on the expression of a reporter gene (such as green fluorescent protein (GFP)) that has been fused to the regulatory sequence of a cardiac-specific gene like MYH6 [213], Nkx2.5 [211], myosin light chain 2 V (MLC2V) [214], or insulin gene enhancer protein 1 (ISL1) [215]. Mitochondrial labeling with a fluorescent dye has also been postulated to be a good selective marker of hESC/hiPSC-derived CMs [204]. Indeed, this approach, combined with FACS, has been shown to generate very highly enriched (> 99% pure) CMs [204]. It is important to note that although more homogenous EBs can be established via massive suspension culture systems, a significant number of iPSCs did not differentiate and thus still carried a strong potential for teratoma formation [205]. Interestingly, in this very study, metabolic purification of CMs using a glucose-depleted and lactate-enriched medium proved to be powerful in eliminating undifferentiated iPSCs, thus generating purer iPSC-derived CMs [205].

It is important to note that a major limiting step for SCT in cardiac regeneration is the purification and enrichment of stem cell-derived CMs. While several approaches for this goal have been employed, their efficiency remains somewhat debatable. There is an agreement, however, that for any such method of purification to be efficient, it ought to be fast, specific, and scalable with no genetic modifications. It is then that such a method can be viewed as a potential therapeutic approach for the use of iPSC-derived CMs in the cardiology clinic.

## Characterization of iPSC-derived CMs: structural and functional properties

Following purification, the iPSC-derived CMs need to be characterized to ensure they have the expected characteristics. The study of structure and function of iPSC-derived CMs is complicated by the fact that the differentiation method [216] and culture conditions [217] may strongly influence phenotype. It is also unclear whether hESC-CMs and iPSC-CMs have different phenotypes. Such method- or cell type-related variation would have significant implications for CM use in both cell therapy and disease modeling. Structure and function in CMs are intimately related and could be assessed using different techniques, including live cell imaging, molecular biology, electrophysiology, and HPLC mass spectrometry (HPLC-MS).

### Live cell imaging

**Fig. 10:**
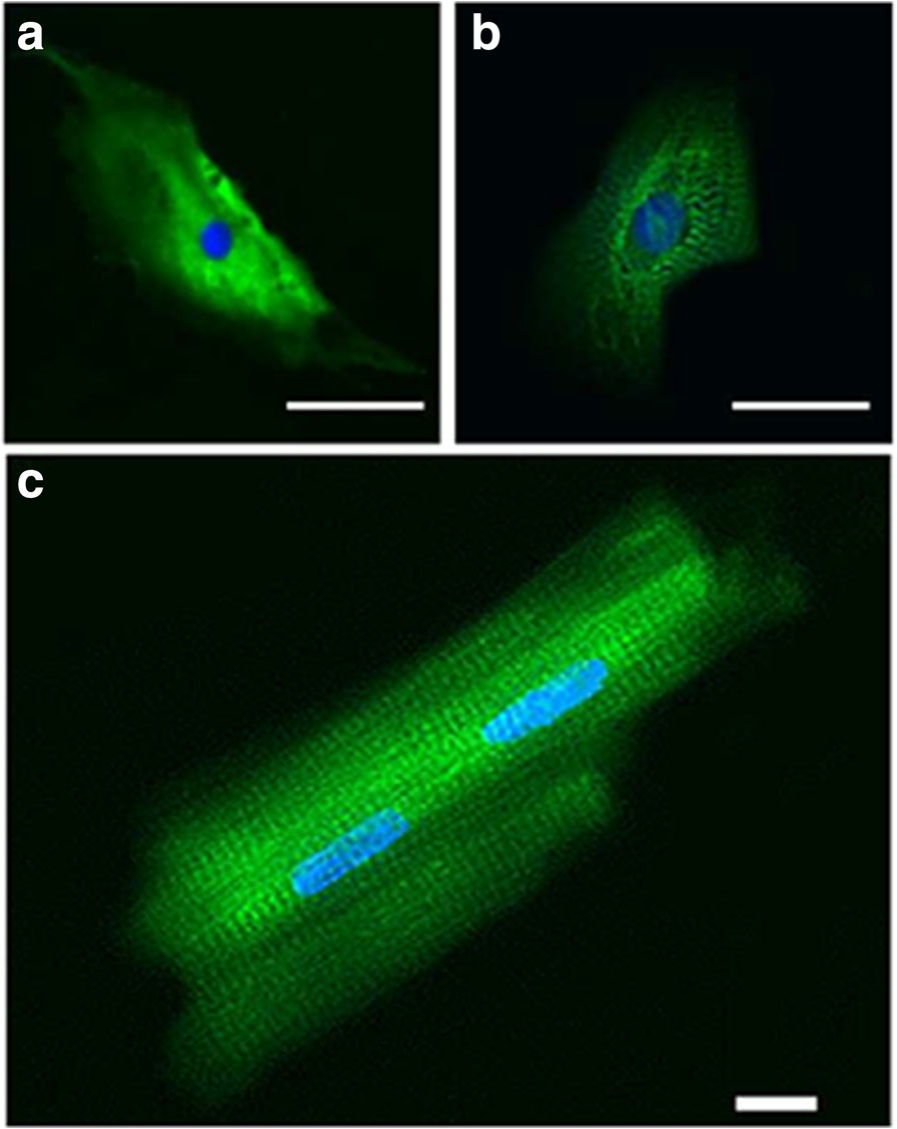
Myosin heavy chain (MHC, *green*) and nuclear (DAPI, *blue*) staining of hESC-CMs without (**a**) and with (**b**) characteristic sarcomeric striation patterns, compared with **c** adult rat ventricular myocyte. *Scale bar* is 20 μm. Figure reproduced with permission of Rao and colleagues. Phenotype and developmental potential of cardiomyocytes from induced pluripotent stem cells and human embryonic stem cells. In: Ainscough J. et al. eds. Nuclear reprogramming and stem cells. Humana Press, 2011 (159). *CM* cardiomyocyte, *hESC* human embryonic stem cell

Live cell imaging yields a large number of cellular measurements that can be used to monitor multiple aspects of cell structure and function. Ultrastructural analysis shows that hESC-CMs develop in vitro from spheroidal cells to elongated cells with a more organized sarcomeric pattern [218] (Fig. 10). Transmission electron microscopy (TEM) of the hESC-CMs at varying developmental stages shows progressive ultrastructural maturation from an irregular myofilament distribution with parallel nascent Z-bands containing myofibrils to a more mature sarcomeric organization containing well-defined sarcomeres with recognizable A, I, and M-bands in older hESC-CMs [217–219]. iPSC-CMs also have functional, albeit immature, sarcomeric structures [220] and comparative studies between hESC-CMs and iPSC-CMs have not shown any difference in ultrastructural phenotype [153]. EM revealed abundant myofibrillar bundles and developed mitochondrial structure in both neonatal mouse CMs and iPSC-CMs. However, iPSC-CMs contained fewer mitochondria with lower density cristae [221]. In addition, Ca^2+^ fluorescent dyes and confocal laser scanning microscopy are commonly used to detect the presence of intact Ca^2+^ handling proteins and assess Ca^2+^ signaling in differentiated CMs [11, 133, 222]. Higher resolution microscopy like two-photon excitation has also been employed to assess the functional coupling (synchronous Ca^2+^ transients) between host and differentiated CMs [223–225]. Recently, Vondriska and colleagues used super resolution stimulated emission depletion (STED) microscopy to investigate chromatin rearrangements in CMs following the induction of cellular hypertrophy [226]. STED imaging techniques can provide spatial resolution that is below the diffraction limit, approaching virtually molecular resolution [227]. They are valuable for the characterization of iPSC-derived CMs and provide novel insights into the structural organization of the differentiated CMs and the dynamics of molecular interactions and cell coupling. Liu and colleagues studied the immunogenicity and rejection of iPSC and iPSC-CM allogenic transplants in murine ischemic myocardium using bioluminescent imaging (BLI) [228]. Their findings revealed that unlike iPSCs, iPSC-CMs and iPSCs differentiated in vivo possess high immunogenicity and are immediately recognized and rejected by the immune system. Immunosuppression stopped this but increased the risk of teratoma formation [228]. Of note, in a separate study, iPSC-CMs efficiently integrated into the healthy myocardium 2 weeks following their transplantation into nude rat hearts [221]. These contradicting findings suggest that iPSC-CM viability and integration into the myocardium might be disease-dependent.

### Molecular biology

Among the different evaluation approaches, molecular biology, immunocytochemistry, qRT-PCR, and phosphoproteomic assays are also used to characterize the functional properties of iPSC-derived CMs. Immunocytochemistry uses antibodies that target specific peptides or protein antigens in the cell via specific epitopes. It is a valuable tool to detect the presence of gap junction proteins (e.g., connexins) at the borders of differentiated CMs where they mediate functional coupling with host CMs [220, 229, 230]. Immunocytochemistry revealed that the major contractile protein in neo-CMs, β-MHC, was similarly expressed in both neonatal mouse CMs and iPSC-CMs, although adhesion molecules such as N-cadherin, α-dystroglycan, and laminin-α2 were less expressed in iPSC-CMs compared to neonatal mouse CMs [221]. Interestingly, transplantation of iPSC-CMs into adult nude rats increased their α-MHC expression, an adult CM-specific molecule, but to a lesser extent than the adult and fetal murine heart [221]. Adhesion molecule protein expression was also detected in iPSC-CMs post-transplantation. These findings strongly support the integration efficiency of iPSC-CMs into the adult myocardium and their capacity to potentially restore myocardial function [221]. In addition, immunocytochemistry has been used to detect the presence of various structural and functional cardiac proteins, such as sarcomeric α-actinin, CTNT, connexin43, α-sarcoglycan, tropomyosin, potassium/sodium hyperpolarization-activated cyclic nucleotide-gated channel 4 (HCN4), Nkx2.5, GATA4, and ANP [153, 221, 230–232]. Quantitative RT-PCR is the next best option to assess the cardiogenic potential of iPSC-derived CMs. It enables reliable detection and quantification of pluripotency and cardiac gene expression levels in differentiated CMs. These data are critical for demonstrating the pluripotency of iPSC lines and for assessing the functionality of CMs. qRT-PCR has been employed in multiple studies to determine the expression levels of stemness genes like OSKM, Nanog, *GDF3*, *REX1*, and *TERT* in iPSCs during their differentiation into functional CMs [11, 12, 153, 233], and similarly in other studies by measuring the expression levels of cardiac genes like Nkx2.5, GATA-4, MEF2C, Tbx5, CTNT, MYH6, α-actinin, myosin light chains (MLCs), myosin heavy chains (MHCs), phospholamban (PLN), ANP, and natriuretic peptide precursor type A (NPPA) [153, 231, 234]. In addition to immunohistochemistry and qRT-PCR, quantitative phosphoproteomic assays have been used to characterize ESC/iPSC functional properties in physiological and pathological conditions [12, 206, 235, 236]. Phosphoproteomics is a branch of proteomics that assesses the phosphorylation of proteins as one of the most important post-translational modifications. Protein phosphorylation acts as a molecular switch to activate or inactivate different proteins. It is a critical event for regulating cellular processes, including cell cycle, growth, differentiation, and signal transduction pathways [237, 238]. During the differentiation process of PSCs, the emerging phosphoproteomic methods enable the deciphering of the cellular signaling that drives cells from pluripotency to specific fates [236, 239, 240].

### Electrophysiology

**Fig. 11:**
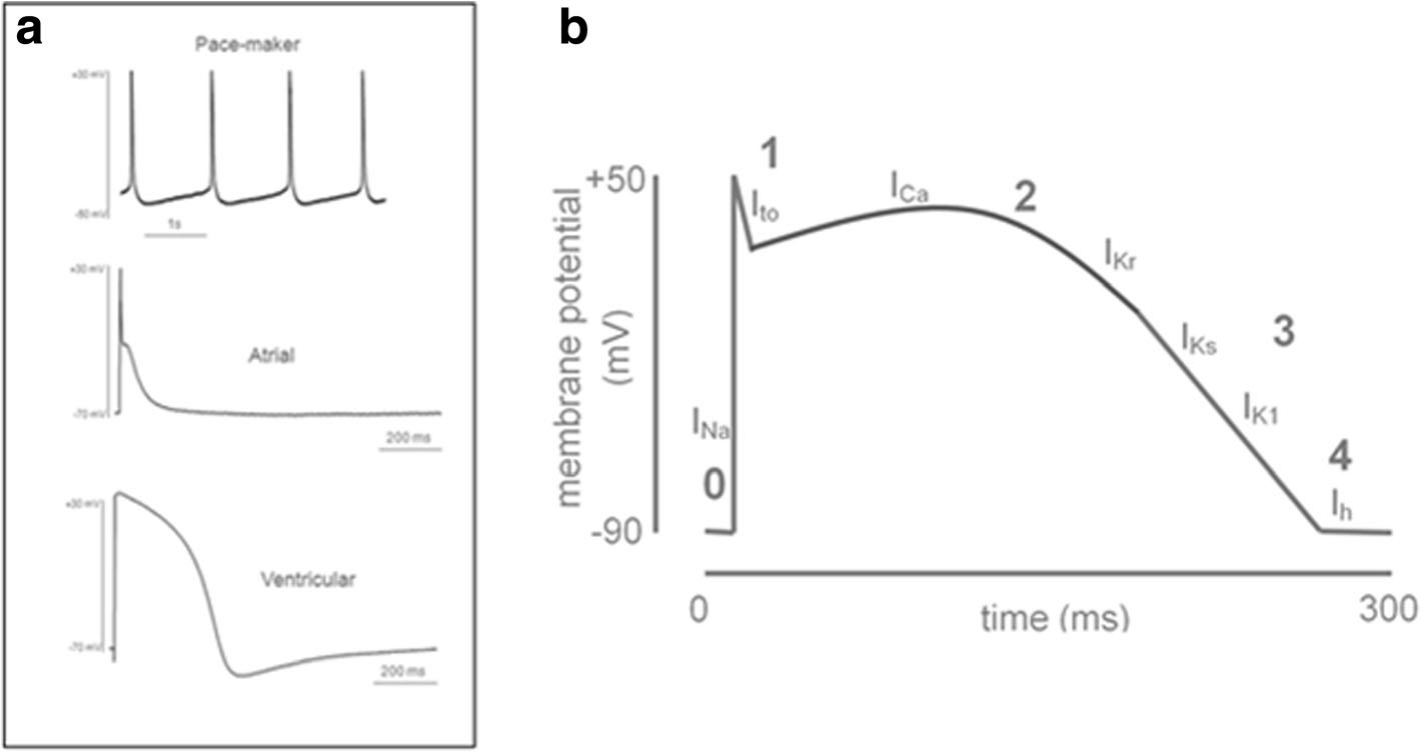
**a** Different action potential phenotypes recorded from hESC-CMs. Figure reproduced with permission of Rao and colleagues. Phenotype and developmental potential of cardiomyocytes from induced pluripotent stem cells and human embryonic stem cells. In: Ainscough J. et al. eds. Nuclear reprogramming and stem cells. Humana Press, 2011 (159). **b** Diagram of an idealized adult human ventricular action potential. The phases of the action potential are labeled (phases 0–4). The predominant cardiac ion currents at each point in the action potential are labeled (*I*_*Na*_ = sodium current, *I*_*to*_ = transient outward potassium current, *I*_*Ca*_ = calcium current, *I*_*Kr*_ = rapidly activating delayed rectifier potassium current, *I*_*Ks*_ = slowly activating delayed rectifier potassium current, *I*_*K1*_ = inward rectifier potassium current). Figure reproduced with permission of Rao and colleagues. Phenotype and developmental potential of cardiomyocytes from induced pluripotent stem cells and human embryonic stem cells. In: Ainscough J. et al. eds. Nuclear reprogramming and stem cells. Humana Press, 2011 (159). *CM* cardiomyocyte, *hESC* human embryonic stem cell, *iPSC* induced pluripotent stem cells

The electrophysiological properties of iPSC and ESC-derived CMs can be characterized using whole cell patch clamp, allowing measurement of action potential (AP) characteristics and specific cell membrane ion currents. Whole cell patch clamp is generally performed on individually isolated CMs. AP characteristics can also be measured from multi-cellular preparations using sharp impalement, or non-invasively using voltage-sensitive dyes. AP characteristics can be inferred from measurement of the field potential by plating individual cells or multi-cellular preparations onto multi-electrode arrays (MEA). MEA and voltage-sensitive dyes also allow AP conduction velocity and propagation patterns in multi-cellular preparations to be measured. AP recorded from iPSC and ESC-derived CMs with morphologies resembling nodal (pacemaker) tissue, atrial, and ventricular tissues have been widely described in the literature. It is unclear if this demonstrates distinct populations of cells committed to differentiation into these three subtypes of mature CMs or simply a population of immature CMs with a heterogeneous phenotype. Cells with “ventricular” AP, for example, often have a high degree of automaticity and an upwards sloping phase 4, which is more typical of nodal cells in adult myocardium. Interestingly, some groups report that the iPSC differentiation method seems to affect the electrophysiological phenotype (Fig. 11). For instance, differentiation protocols based on EBs lead to equal numbers of ventricular- and atrial-like cells whereas the END-2 co-culture method results in homogeneous populations of ventricular-like cells [154]. Pharmacologically, several groups reported that iPSC- and ESC-derived CMs have similar responses to pharmacological agents as adult CMs, suggesting expression of ion channels and key receptors resembling adult CMs. In particular, pharmacological blockade of the rapid delayed rectifier potassium (I_kr_) channels results in the prolongation of the AP duration in ESCs and iPSC-derived CMs, whilst blockade of calcium channels results in the shortening of the AP duration [10, 216, 217, 220, 232, 241]. Chronotropic responses to adrenergic stimulation have also been recorded [10, 147, 151, 216, 220, 232].

### HPLC-mass spectrometry

Structural changes following cellular differentiation can be assessed using analytical chemistry methods. Kawamura and colleagues analyzed N-glycan transition during iPSC-CM differentiation using HPLC-MS methods [242]. Cell surface glycans are functional proteins with multiple roles, including cell–cell adhesion, cell activation, and cellular response to growth and arrest. Expression patterns of cell surface glycans change during differentiation as shown in ESCs [243]. In their study, Kawamura and colleagues isolated 68 different N-glycans and identified the structures of 60 of these proteins. Isolated N-glycans were analyzed based on their HPLC elution positions and MALDI-TF/MS. Findings showed structural differences between iPSCs, iPSC-CMs, and mouse myocardium. Decreases in high mannose and neutral N-glycans versus increases in focusylated, monosialyl, and disialyl N-glycans were observed when comparing iPSCs to iPSC-CMs to mouse myocardium sequentially. Additionally, some structural differences were detected between iPSC-CMs and mouse myocardium. The murine myocardium was especially rich in NeuGc-type sialyl structures, which corresponded to cytidine monophosphate-N-acetylneuraminic acid hydroxykase (CMAH) expression that was relatively limited in the heart. iPSC-CMs also expressed several unique glycans with Galα1-6Gal structure [242]. The pattern of N-glycan distribution revealed in this study could be used as a platform for future investigations in order to define markers of maturity following iPSC-CM transplantation into the myocardium.

In summary, iPSC- and ESC-derived CMs appear to resemble the biochemical and molecular signatures of adult CMs, along with some of their structural and functional properties. However, some iPSC- and ESC-derived CMs retain the phenotype of immature myocytes. Whether this affects their utility in regenerative medicine or as disease models is not apparent and will be further discussed in the following sections.

## Cardiovascular disease modeling

Disease modeling is an integral component of research efforts to understand the pathogenic mechanisms of CVDs and unveil promising therapeutic targets. Although large and small animal models have been extensively used for modeling human CVDs, they are expensive, ethically problematic, and their contribution to understanding human disease is arguably limited by their fundamental biological differences. hESCs, whilst also ethically problematic, have also been explored as CVD models by introducing causative human gene mutations into hESCs and inducing their differentiation into functional CMs in vitro. iPSCs have significant advantages over ESCs as they are derived from somatic cells, circumventing most ethical objections to ESC technology, and carry genetic mutations as well as any other modifier genes and contributing genetic factors, potentially facilitating recreation of patient-specific disease phenotypes in vitro.

However, literature shows that iPSC-derived CMs are structurally, functionally, and genetically similar to early embryonic CMs [244]. Despite maturation in culture, these cells are arrested at a phase corresponding to the late embryonic/early neonatal stage [245]. This could potentially mask a disease phenotype due to differential expression of proteins with interfering or modulatory functions [246]. As well, with most cardiomyopathic manifestations appearing in adulthood, it becomes imperative to direct iPSC-derived CMs to complete maturation. To this end, studies have used mechanical and electrical stimulation approaches in vitro to promote structural and functional maturation [247–249]. Others used varied culture substrate/arrangement to enhance maturation [250, 251]. In addition, work with native cardiac extracellular matrix in a 3D culture system improved iPSC-derived CM maturation [252]. A recent study used a different approach to obtain mature human iPSC-derived CMs [253]. Human iPSCs were differentiated in vitro into cardiac progenitor cells that were later transplanted into rat neonatal hearts. Within one month of transplantation, these cells developed into adult CMs and revealed patient-specific disease phenotype.

### Modeling long-QT syndromes

iPSCs were first used to replicate a cardiovascular disease phenotype in vitro by Moretti and colleagues [10]. They compared wild-type cells with patient-specific iPSC-CMs containing an inherited autosomal dominant [596G→A] missense mutation in the KCNQ1 gene associated with LQTS1. Using single-cell patch clamp assays, the authors found that patient-specific iPSC-CMs displayed prolonged atrial and ventricular APs and reduced repolarization velocity compared to wild-type cells. Furthermore, electrophysiological analysis showed reduction in the slow outward potassium current (I_ks_) in ventricular patient-specific iPSC-CMs compared with controls (Table 2). In contrast to wild-type cells, β-adrenergic stimulation of ventricular patient-specific iPSC-CMs with isoproterenol had no significant effect on the repolarization and I_ks_ currents (Table 2). Additionally, immunocytochemical analysis of patient-specific CMs showed impaired protein trafficking and membrane delivery which correlated with the disease phenotype. Two other studies modeled LQTS2 using similar methods. Itzhaki and colleagues derived iPSC-CMs from a patient with LQTS2 containing a missense mutation in the KCNH2 gene, which affected the delayed rectifier potassium channel (I_kr_) [229]. The derived iPSC-CMs displayed the electrophysiological abnormalities of the disease, including prolonged AP duration and reduced repolarization velocity. As expected, the I_kr_ was significantly reduced in patient-specific cells in which an increased sensitivity to arrhythmogenic agents was detected. The authors further tested the therapeutic effect of nifedipine (antihypertensive), pinacidil (vasodilator), and ranolazine (antianginal) on the electrophysiological properties of diseased iPSC-CMs. The drugs were found to shorten AP duration and abolish abnormal depolarization (early after depolarization (EAD)). Similarly, Matsa and colleagues successfully derived CMs from related LQTS2 patients with KCNH2 mutation [13]. Using patch clamp and microelectrode array mapping, the authors demonstrated that LQTS2 iPSC-CMs displayed prolonged APs and corrected field potential duration (cFPD). The authors tested the effect of E-4031 (antiarrhythmic) on patient-specific iPSC-CMs and found an elongation of AP duration and induction of EAD. In addition, application (individually or together) of nicorandil (vasodilator) and PD-118057 (antiarrhythmic) was found to shorten AP duration and abolish EAD (nicorandil).

**Table 2:** Patient-specific iPSC-CMs in cardiac disease modeling

Disease modeled	Genetic disorder	Phenotypical assessment	iPSC-CM abnormality	Patients	Control	Reference(s)
LQTS-1	KCNQ1	Patch clamp Immunohistochemistry	I_ks_ decrease Adrenergic response	2	2 healthy individuals	[10]
LQTS-2	KCNH2	Patch clamp Electron recording Pharmacology	I_ks_ decrease APD prolongation	1	1 healthy individual	[229]
LQTS-2	KCNH2	Patch clamp Microscopy	APD prolongation Drug sensitivity increase	2	CMs from HUES7 cell lines and genetically unrelated hESC-derived fibroblasts	[13]
LQTS-8 (Timothy syndrome)	CACNA1C	Patch clamp	I_Ca_	2	2 healthy individuals	[11]
Leopard syndrome (HCM)	PTPN11	Microscopy Immunocytochemistry Western blotting Antibody array	Large cells, high degree of sarcomeric organization, preferential nuclear localization of NFATC4	2	hESCs and 1 healthy individual	[12]
DCM	TNNT2	Patch clamp Electrode recordings Microelectrode array Atomic force microscopy	Altered Ca^2+^ handling Decreased contractility, abnormal sarcomeric organization, increased susceptibility to adrenergic stimulation and bio-mechanical stress	Many	Many healthy individuals	[257]

*ADP* action potential duration, *DCM* dilated cardiomyopathy, *hESC* human embryonic stem cell, *iPSC-CM* induced pluripotent stem cell-derived cardiomyocyte, *I*_*kr*_ delayed rectifier potassium current, *I*_*ks*_ slow outward potassium current, *LQTS* long QT syndrome

Yazawa et al. [11] have also successfully recreated the LQTS phenotype in iPSC-derived CMs generated from patients with Timothy syndrome (LQTS8). This disease, characterized by a mutation in the CACNA1C gene encoding the subunit Cav1.2 of the voltage-gated calcium channel in humans, results in multi-system abnormalities including LQTS [11]. Recently, Liew and colleagues successfully generated iPSC-CMs from a patient with arrythmogenic right ventricular cardiomyopathy associated with plakophylin-2 (PKP2) mutation and are in the process of modeling the disease in vitro [133].

Another study examining the same disorder used iPSC-CMs to uncover the role of a different mutation in the sodium channels [246]. Similar techniques were employed to provide a model for catecholaminergic polymorphic ventricular tachycardia that was useful to evaluate the therapeutic potential of a ryanodine receptor ligand [254]. On the other hand, Okata and colleagues successfully showed that the LQTS3 phenotype is recapitulated by a SCN5A sodium channel mutation that was maintained in hiPSCs derived from a Brugada syndrome patient, yet the Brugada syndrome phenotype was not displayed until SCN5B expression, increased due to the embryonic nature of these cells, and was opposed by knock-down [246].

One of the technical problems encountered in this type of cellular assay is the phenotypic heterogeneity of the derived CMs between atrial, ventricular, and nodal cells, which express different AP patterns early after depolarizations. A recent study [255] proposed the use of a genetically encoded membrane voltage sensor with promoters that drive its expression in hiPSC-CMs to select the relevant cell types to use for drug screening.

### Modeling inherited cardiomyopathies

Arguably, existing experimental tools are sufficient to model single ion channel disorders, and consequently the challenge is to leverage the potential of iPSCs to model more complicated disease phenotypes. One of the earliest attempts to do this was for the LEOPARD syndrome an autosomal dominant multisystem disorder resulting from a missense mutation in the PTPN11 gene that results in abnormalities of the skin, skeletal muscle, and cardiovascular system [12]. The most commonly life-threatening cardiac anomaly associated with LEOPARD syndrome is hypertrophic cardiomyopathy (HCM). Carvajal-Vergara and his colleagues showed that compared to control iPSC lines, iPSC-CMs from a LEOPARD syndrome patient had a higher mean cell surface area, a greater degree of sarcomeric assembly, and a nuclear localization of the NFATC4 transcription factor. In addition, phosphoproteomic assays of these CMs revealed a notable abundance or increased phosphorylation of proteins that could be involved in the cardiac hypertrophy observed in these patients. Although they were unable to fully characterize the observed hypertrophic phenotype because of the heterogeneity of the iPSC-derived CM population, they were able to suggest novel molecular mechanisms that may underlay the development of the hypertrophic phenotype in this patient population, supporting the utility of iPSC-CMs as a disease model. iPSC lines were created from a family with isolated familial HCM who carried a missense mutation in the MYH7 gene. Despite mutations of genes encoding sarcomeric proteins being the classic cause of familial HCM, the mechanisms that lead to the development of the HCM phenotype is unclear. This study was able to replicate the HCM phenotype at the cellular level, showing cellular, contractile, and electrophysiological enlargement [256]. Unlike the aforementioned study, the authors were also able to demonstrate activation of a hypertrophic gene expression pattern; significantly, however, this was achieved using single-cell gene expression analysis, negating the effect of population heterogeneity. Not only were the authors able to demonstrate that deranged calcium hemostasis was critical to the development of the HCM phenotype, but pharmacological normalization of calcium hemostasis was able to prevent the development of the HCM phenotype, suggesting novel therapeutic mechanisms [256]. Similarly, iPSC lines have been generated from a family with familial dilated cardiomyopathy (DCM), caused by a mutation of the gene encoding cardiac troponin T (TNNT2) [257]. iPSC-derived CMs differentiated from patients with DCM exhibited a DCM phenotype with deranged sarcomeric organization, altered calcium handling and increased susceptibility to biomechanical stress and adrenergic stimulation [257]. The authors found that β-blockade and Serca2a overexpression partially normalized the adverse phenotype observed in DCM iPSC-CMs [257].

A further study, which created iPSC-CMs from DCM patients, demonstrated a different application from the previous studies for iPSCs in cardiovascular disease modeling [258]. In this study recreation of the DCM cellular phenotype using iPSC-CMs from a patient with a novel mutation of the gene encoding desmin was used to support the assertion that this mutation was responsible for the development of the DCM phenotype in this patient [258].

Despite promising evidence that complex cellular phenotypes can be modeled using iPSC-CMs, questions remain. Several of these studies suggest that there was heterogeneity in the population of iPSC-CMs, or inclusion of non-CMs following the use of common differentiation techniques. This may limit the utility of iPSC-CMs for multicellular assays, which are the mainstay of molecular biological and cellular physiology, and consequently limit their utility as disease models. Some studies were able to recreate subcellular phenotypes in iPSC-derived CMs whilst failing to recreate DCM or HCM cellular phenotypes, suggesting that culture conditions or cell–cell interaction may be critical in developing disease phenotypes [259, 260]. Overall, it remains to be shown whether the cellular defects evident can be modeled in a meaningful fashion using cells with such an immature and heterogeneous phenotype.

## Drug screening and development

Drug development is expensive and high-risk [261, 262], with the average cost for a drug being estimated to exceed US $800 million [262]. This can be largely attributed to the large number (approximately 80%) of chemical compounds which are rejected at some stage during clinical trials [262]. In half of cases this is due to reduced efficacy, and in the other half to increased toxicity (commonly cardiac or hepatic toxicity) [14]. Cardiac toxicity can lead to reactive oxygen species formation, altered contractility, arrhythmia, impaired gene expression, and cell death. The current models used in the pharmaceutical industry for cardiac drug toxicity screening rely on animal CMs, immortalized human cell lines, and animal models. Although these models provide useful information in evaluating the safety and efficacy of the drugs, they fail to replicate human pathophysiological conditions. The development of reliable in vitro models for drug screening and toxicity is a major challenge in drug development. iPSC-CMs from patients with a range of genetic backgrounds and disease phenotypes could potentially provide a high-throughput platform for toxicology screening and drug development [153, 263]. A recent study used transcriptome profiling of iPSC-CMs to verify that reprogramming preserved patient-specific patterns of metabolic and stress-response gene expression [264]. The transcriptome-based toxicology analysis in this study predicted cardiotoxicity in a manner that was concordant with functional assays used for validation. However, many of the caveats that apply to the use of iPSC-CMs as disease models apply to their use as toxicology screening and drug development tools. As iPSC-CMs have an immature and heterogeneous phenotype, experimental findings will need to be considered in conjunction with, rather than instead of, existing animal models.

## Cardiac regeneration

Current therapies for heart failure are largely palliative, aiming to prevent the progression of heart failure and relieve symptoms [3]. The only treatment for end-stage heart failure with established long-term efficacy is cardiac transplantation [4]. The increasing prevalence of heart failure and existing shortage of donor organs makes transplantation an unsustainable long-term solution [5]. Consequently, there is a major need to develop novel therapeutic strategies.

### Myocardial repair

Cell therapy entails either mobilization of endogenous cardiac progenitor cells or transplantation of exogenous stem cells. Interestingly, these therapies are not mutually exclusive, and it has been widely suggested that cell transplantation promotes mobilization of endogenous stem cells [265]. Several cell types have been suggested for use in cardiac regeneration, including skeletal myoblasts, bone marrow-derived stem cells, endothelial stem cells, MSCs, and cardiac stem cells (CSCs) [3, 29, 133, 266–272] (Table 3). All cell types appear to induce a transient improvement in cardiac physiology in humans and animal models. However, it is now considered unlikely that this can be explained by induction of myogenesis alone [273]. Improvements in function were often before significant myogenesis could have occurred, suggesting that functional improvements in the existing cells are responsible [274]. Improvements in cardiac physiology were seen irrespective of cell type [275] and delivery method and without an expected dose effect [276]. Despite promising early reports, there has been a paucity of evidence demonstrating the presence of new CMs is significant enough to account for even these moderate, transient improvements in cardiac function [277, 278]. A view based on some recent observations might offer an explanation via a role for exosomes containing angiogenic factors released from these cells in a paracrine manner [279]. Indeed, several studies have proposed the use and delivery of exosomes derived from iPSC-CMs to improve cardiac function in animal models [280].

**Table 3:** Characteristics of different types of stem cells for cardiac regeneration

	BMSCs	MSCs	MBs	CSCs	EPCs	ESCs	iPSCs
Origin	Bone marrow	Bone marrow, heart, lung, adipose tissue	Muscle	Heart	Bone marrow, peripheral blood	ICM of blastocysts	Diverse tissues
Differentiated cells							
CMs	Controversial	Controversial	Controversial	Possible	Impossible	Possible	Possible
Endothelial cells	Possible	Possible	No report	Possible	Possible	Possible	Possible
SMCs	Possible	Possible	No report	Possible	Impossible	Possible	Possible
Other cell types	Unknown	Possible	No report	Controversial	Impossible	Possible	Possible
Immunogenicity	Unlikely if autologous	Unlikely if autologous	Unlikely if autologous	Unlikely if autologous	Unlikely if autologous	Exists	Unlikely if autologous
Electrochemical coupling	No	Yes	No	Possible	–	Possible	Possible
Paracrine effect	Exists	Exists	Exists	Exists	Exists	Exists	Exists
In vitro expansion	Limited	Limited	Limited	Limited	Limited	Yes	Yes
Clinical safety	Yes	Yes	Side effects reported	Yes	Yes	Teratoma formation, ethical concerns, arrhythmia	Teratoma formation, mutagenesis, arrhythmia

*BMSC* bone marrow stem cell, *MSC* mesenchymal stem cell, *MB* mybolast, *CSC* cardiac stem cell, *EPC* endothelial progenitor cell, *ESC* embryonic stem cell, *ICM* inner cell mass, *iPSC* induced pluripotent stem cell, *CM* cardiomyocyte

Most of the clinical experience had been with autologous CD34+ cell transplantation [281, 282]. These cells were either obtained by bone marrow aspiration or purified from peripheral blood following mobilization with G-CSF. These cells received no treatment or modulation prior to injection into patients. Collective clinical evidence shows that, following percutaneous coronary intervention, transplantation of these cells reduced long-term mortality, development of arrhythmias, and non-fatal myocardial infarction (MI). However, there was no improvement observed in ejection fraction or hospitalization due to heart failure.

ESCs and iPSCs, in contrast to many of the multipotent cell types used in clinical trials and animal models, are pluripotent and can differentiate into all cell types in the body, including CMs [78, 153, 230, 283–286]. Furthermore, unlike CSCs, which also have CM differentiation potential, iPSCs and ESCs can be expanded and cultured for many months without loss of phenotype [287]. Furthermore iPSCs theoretically facilitate allogeneic transplantation [288, 289].

Researchers have reported that transplantation of ESC-CMs into the infarcted hearts of rodents improve cardiac function [148, 149, 290–294]. Similarly, iPSCs have been shown to improve cardiac function in rodent models of MI [267, 295]. The engrafted iPSCs were able to restore contractile performance, attenuate pathological vascular remodeling, and enhance electrophysiological properties, while also achieving in situ cardiac tissue regeneration. Three-dimensional human heart constructs consisting of hiPSC-CMs and endothelial cells were used to repair large cardiac defects. These constructs markedly improved cardiac function, with increased myocyte proliferation, vascularization, and electrical coupling to the intact heart [296]. Similar regenerative potential was also seen when hiPSC-CMs were used to seed biodegradable tissue grafts used to repair ventricular defects in rats, indicating a potential use in congenital defects [297].

Novel potential applications of iPSC technology involve their use in combination with the zinc-finger nuclease (ZFN) technique to genetically modify patient-specific iPSCs [298]. This technique involves using genetically engineered ZFNs to cleave DNA sequences (containing the disease-causing mutations), allowing the endogenous DNA repair machinery to make targeted gene correction or “genome editing”. The ZFN technique was originally used for manipulating the genomes of many plants and animals, but has recently been applied to iPSCs for treating genetic abnormalities responsible for α-1 antitrypsin deficiency [299] and sickle cell anemia [300]. Similarly, this approach could be adapted to correct genetic mutations in patient-derived iPSC-CMs, so they can be implanted back into the patients, avoiding the need for immunosuppression. Additionally, CRISPR/Cas-9 was successfully applied on iPSC-CMs from patients with arrhythmogenic right ventricular cardiomyopathy showing atypical sodium channel mutations [301]. The corrected myocytes showed normal channel activity and expression. However, these techniques are at an early stage and require extensive investigation to ensure the necessary accuracy, efficiency, and safety prior to applying them in clinical practice.

### Cardiac pacing

iPSCs have also been proposed as “biological pacemakers” in patients with acquired arrhythmia. The contraction of heart muscle is initiated by a syncytium of specialized cells (pacemaker cells) responsible for the generation of rhythmic impulses that control heart rate, which are propagated through the myocardium through specialized conducting fibers [302]. This mechanism can fail following ischemic insult or as a result of degenerative heart disease, often requiring insertion of an artificial cardiac pacemaker to restore cardiac function. The development of “biological pacemakers” can bypass the need for implantable pacemakers and its associated risks, including immune rejection, infection, and generator/battery replacement. One of the original reports about the use of hESCs for cardiac pacing found that hESC-CMs were capable of pacing the hearts of swine with complete atrioventricular block and restoring the electromechanical properties of the myocardium [303]. Similar results have been obtained using genetically engineered hESCs transplanted into guinea pig hearts [304]. iPSCs are potentially preferential to hESCs for the same reasons discussed in the previous section relating to myocardial regeneration; however, in addition to the tumorigenicity and immunogenicity of iPSCs, numerous technical problems exist before this potential application can be translated to the clinic [302]. The immature pacemaker mechanisms in iPSC-CMs and their heterogeneous phenotype, for example, may make them potentially dangerous artificial pacemakers.

iPSC-based cell therapy is not, however, without technical difficulties and these will need to be overcome before they can be readily applied in clinical practice. Firstly, reports suggest that undifferentiated iPSCs may elicit a significant host immune response [83]. Whilst this appears to be overcome by differentiating the cells into the host tissue lineage before injection, this may potentially negate many of the benefits of using a pluripotent stem cell line [305, 306]. Nevertheless, a recent study reported promising results with a microRNA switch designed to detect and remove hiPSCs and partially differentiated cells, thus preventing teratoma formation, before transplantation [307]. Furthermore, long-standing concerns about the tumorigenicity of iPSCs relate to both the activation of oncogenes during reprogramming and their pluripotent nature [308]. Novel reprogramming technology that avoids viral–genome integration and injection of differentiated cells may overcome this problem; however, it requires that reprogramming, expansion, differentiation, and CM purification protocols be optimized before iPSC technology can be brought into the clinic [308].

### Beyond iPSC technology: direct reprogramming of fibroblasts into functional CMs

Srivastava and colleagues extended the concept of cellular reprogramming by demonstrating direct trans-differentiation of murine fibroblasts into functional CMs by retroviral delivery of three cardiac transcription factors: Gata4, Mef2c, and Tbx5 (GMT) [231]. One week after transduction, expression of cardiac troponin T, sarcomeric α-actinin, and atrial natriuretic peptide was detected in ~ 30% of the cells. Injected into murine hearts, these three factor-reprogrammed cells showed rapid differentiation into CMs that were epigenetically and functionally similar to wild-type CMs. However, CM-specific genes like actin-α cardiac muscle-1 (ACTC1), myosine-6 (MYH6), ryanodine receptor-2 (RYR2), and gap junction α-1 (GJA1) were not expressed. Recently the same group demonstrated that resident cardiac fibroblasts in the murine heart can be reprogrammed into CMs by local delivery of GMT-loaded retrovirus after coronary ligation [309]. The induced CMs infiltrated into the infarct border zone, electrically matured, and coupled with the endogenous CMs. Using genetic lineage tracing, the authors showed that these induced CMs were descendants of cardiac fibroblasts. Moreover, the in vivo delivery of GMT decreased infarct size and modestly improved cardiac function up to 3 months after coronary ligation.

A modified protocol has been used to directly reprogram mouse embryonic fibroblasts (MEFs) into beating CMs without an intermediate pluripotent state [91]. Efe and colleagues showed that a brief overexpression of OKSM factors (~ 4 days) in conditioned media could efficiently generate spontaneously contracting patches of CMs over a period of 11–12 days. Importantly, the application of the small-molecule JAK/STAT pathway inhibitor JI1 primes the transient pluripotent cells towards a cardiac fate. This observation strongly supports a role for JAK/STAT signaling in the regulation of cardiac cell differentiation [310].

These studies suggest that differentiated somatic cells can be reprogrammed directly into functional CMs without transitioning first to an intermediate pluripotent state. Bypassing the pluripotent stage not only potentially makes generation of reprogrammed CMs much more efficient but significantly reduces the tumorigenic risk associated with the use of pluripotent stem cells. Nevertheless, these strategies still rely on viral vectors for reprogramming, which raises safety concerns and may limit their application in clinical practice.

## Conclusions and futures perspectives

**Fig. 12:**
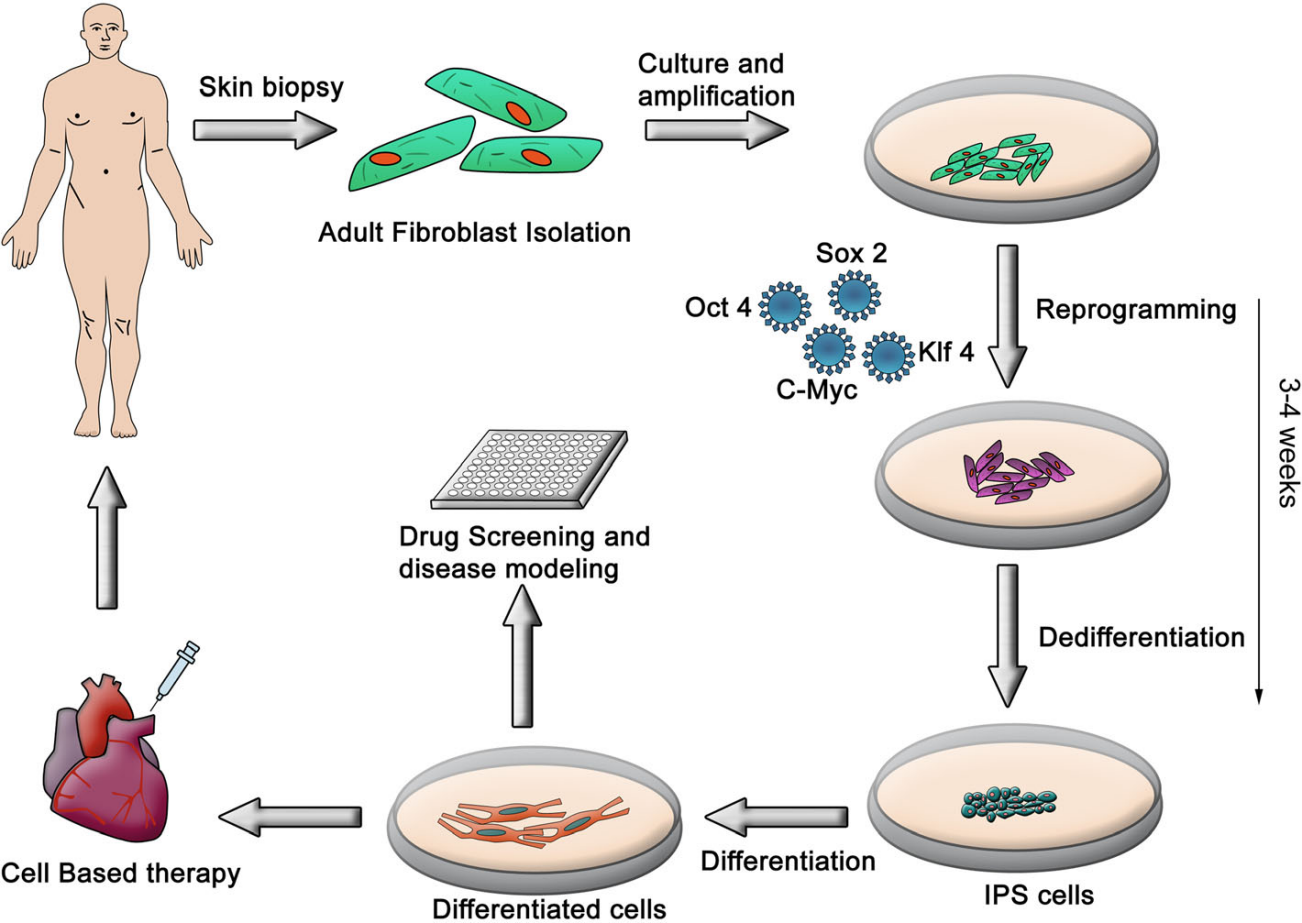
Application of hiPSC technology in cardiovascular medicine. Fibroblasts can be obtained from skin biopsies and derived into iPSCs in vitro as previously discussed. iPSC differentiation into CMs allows the study of the cellular and mechanical aspects of a variety of genetic diseases. In vitro drug screening for reversion of the particular affliction can be tested on such “diseased” CMs. When derived from healthy donors, iPSCs and CMs can be used to test the cardiac toxicity of drugs. The use of “healthy” iPSC-CMs for cellular therapy is also considered as a potential application of iPSC technology in regenerative medicine. *CM* cardiomyocyte, *iPSC* induced pluripotent stem cell

Over the course of only a few years since Yamanaka’s pioneering work on cellular reprogramming, the field of iPSCs has evolved dramatically. The lessons previously learned from ESCs enabled the rapid application of iPSCs to CVD modeling and cell therapy (Fig. 12). The challenge now lies in translating iPSC technology to the clinic in a safe and effective manner. This will require, among other steps, significant optimization of the reprogramming, cell expansion, differentiation, purification, and characterization protocols. Given the rapid developments in this field and the heightened international focus on iPSCs, significant advances are expected in the coming years in delineating CVD genotype–phenotype associations, depicting new therapeutic targets, assessing new drugs faster and more effectively, as well as designing cell- or combined gene–cell or drug–cell therapies. Once optimized, these therapeutic tools may potentially transform clinical practice and ultimately the quality of life and life expectancy of patients with CVDs.

## Abbreviations

AP: Action potential
ASC: Adult stem cells
BLI: Bioluminescent imaging
cFPD: Corrected field potential duration
CM: Cardiomyocyte
CSC: Cardiac stem cell
CVD: Cardiovascular disease
EAD: Early after depolarization
EBNA-1: Epstein-Barr nuclear antigen-1
ECC: Embryonal carcinoma cell
EMILIN2: Elastin microfibril interface 2
ESC: Embryonic stem cells
GMT: Gata4, Mef2c, and Tbx5
HCM: Hypertrophic cardiomyopathy
hEF: Human embryonic fibroblast
HEK293: Human embryonic kidney 293
hiPSC: Human iPSC
HSC: Hematopoietic stem cell
ICM: Inner cell mass
iPSC: Induced pluripotent stem cell
ITRs: Inverted terminal repeat
LQTS: Long QT syndrome
MEA: Multi-electrode arrays
MI: Myocardial infarction
miPSC: Mouse iPSC
MLC2V: Myosin light chain 2 V
NIH: National Institutes of Health
OSKM: Oct3/4, Sox2, Klf4, and c-Myc
PDGFR-α: Platelet-derived growth factor-α
PSC: Pluripotent stem cell
SCNT: Somatic cell nuclear transfer
SCT: Stem cell therapy
SIRPA-α: Signal regulatory protein-α
STED microscopy: Super resolution stimulated emission depletion microscopy
SVLT: SV40 large T antigen
TEM: Transmission electron microscopy
VCAM1: Vascular cell adhesion protein1
VEGFR2: Vascular endothelial growth factor 2
VPA: Valproic acid
ZFN: Zinc-finger nuclease
